# Wound fluid sampling methods for proteomic studies: A scoping review

**DOI:** 10.1111/wrr.13009

**Published:** 2022-04-05

**Authors:** Joe Harvey, Kieran T. Mellody, Nicky Cullum, Rachel E. B. Watson, Jo Dumville

**Affiliations:** ^1^ Centre for Dermatology Research, School of Biological Sciences The University of Manchester & Salford Royal NHS Foundation Trust, Manchester Academic Health Science Centre UK; ^2^ NIHR Manchester Biomedical Research Centre, Manchester University NHS Foundation Trust, Manchester Academic Health Science Centre Manchester UK; ^3^ Division of Nursing, Midwifery & Social Work School of Health Sciences, The University of Manchester Manchester UK; ^4^ Manchester Institute for Collaborative Research on Ageing The University of Manchester Manchester UK

**Keywords:** complex wound, surgical wound, wound biomarker, wound fluid collection, wound proteomics

## Abstract

Understanding why some wounds are hard to heal is important for improving care and developing more effective treatments. The method of sample collection used is an integral step in the research process and thus may affect the results obtained. The primary objective of this study was to summarise and map the methods currently used to sample wound fluid for protein profiling and analysis. Eligible studies were those that used a sampling method to collect wound fluid from any human wound for analysis of proteins. A search for eligible studies was performed using MEDLINE, Embase and CINAHL Plus in May 2020. All references were screened for eligibility by one reviewer, followed by discussion and consensus with a second reviewer. Quantitative data were mapped and visualised using appropriate software and summarised via a narrative summary. After screening, 280 studies were included in this review. The most commonly used group of wound fluid collection methods were vacuum, drainage or use of other external devices, with surgical wounds being the most common sample source. Other frequently used collection methods were extraction from absorbent materials, collection beneath an occlusive dressing and direct collection of wound fluid. This scoping review highlights the variety of methods used for wound fluid collection. Many studies had small sample sizes and short sample collection periods; these weaknesses have hampered the discovery and validation of novel biomarkers. Future research should aim to assess the reproducibility and feasibility of sampling and analytical methods for use in larger longitudinal studies.

**Abbreviations**
BRC: Biomedical Research Centre
TIDieR: Template for Intervention Description and Replication

## 1 INTRODUCTION

### 1.1 Rationale

Wounds are disruptions to the integrity of the skin and may compromise its structure and function, depending on wound severity.^1^ Wounds can be classed as open or closed, with closed wounds having their edges bought together and held (e.g. with stiches) and open, or complex, wounds left to heal by secondary intention. Complex wounds, which include leg, foot, and pressure ulcers, can take months to heal and in some cases will not heal. The treatment of complex wounds is typically time‐ and resource‐intensive, and their chronicity can be distressing for those directly affected.^2^, ^3^, ^4^ Biomarkers can give an indication of a person's biological state and may be useful for understanding, or predicting, the healing trajectory of a wound.^5^ Proteomic profiling of the wound micro‐environment is one avenue for possible identification and validation of biomarkers^6^ as well as being useful for uncovering the potential mechanism(s) of delayed healing.^7^ Therefore, investigating the proteomic profile of the wound environment has become an important focus in wound research.^8^, ^9^, ^10^

There are a number of sample types, which are collected for investigations of the wound environment. Wound fluid is commonly used for protein profiling and analysis^11^ and has many characteristics that make it an ideal sample type for biomarker identification.^12^, ^13^ Although there are multiple methods of collecting wound fluid for research purposes, there has been no comprehensive overview, which clearly presents their details and pattern of use. Furthermore, a large dataset, which numerically summarises the collection methods used for wound healing research, may be useful to ascertain whether more stringent guidelines are required for standardisation of the current methods.^14^ Therefore, a scoping review was performed to systematically map the methods used for wound fluid collection and analyses and to identify whether any gaps in the research base existed.

### 1.2 Objectives

The primary objective of the scoping review was to answer the question:

1. Which sample collection methods have been used to collect wound fluid for the analysis of protein expression or activity?Three related sub‐questions were further addressed as follows:

a. What study designs and sampling regimens have been used (number of samples and frequency of sampling)?

b. How were wound fluid samples processed and stored before proteomic analysis?

c. Which proteomic analysis methods have been used to analyse wound fluid, and what is the relationship between collection and analysis methods?

## 2 MATERIALS AND METHODS

A protocol was first developed outlining how the scoping review would be carried out. This protocol was registered with the open science framework on 27 May 2021 (https://osf.io/5qywx). The scoping review was reported with reference to the PRISMA extension for scoping reviews (PRISMA‐ScR) checklist^15^ (Appendix S1).

### 2.1 Study inclusion criteria

Studies were eligible for inclusion in this review if they used a sampling method to collect wound fluid from any human wound for analysis of proteins or for proteomics (including endogenous protein expression/activity and expression/activity of their inhibitors). No restrictions were applied to study design, sample size, publication date or participant characteristics. Studies of other tissue types, e.g., from biopsy, were excluded. Only primary publications were accepted; however, reviews identified by the initial search were screened for potentially relevant studies. Finally, only full‐text articles written in, or available in, the English language were included in this review.

### 2.2 Search strategy

Three separate databases: Ovid (MEDLINE), Ovid (Embase) and EBSCO (CINAHL plus) were searched on 21 May 2020. The search strategy was developed by an information specialist and the search terms selected based on the above inclusion criteria. This strategy allowed for inclusion of a broad range of studies that could then be screened for relevance. The search was initially split into two separate categories: open wounds and surgical wounds. The initial search strategy used for each database, including all search terms, is outlined in the supplementary material along with the relevant search results (Appendix S2).

### 2.3 Screening

All articles were de‐duplicated in Endnote X9 (Clarivate Analytics, Philadelphia, PA, USA) and the resulting citations were uploaded to review software (Covidence; Covidence, Melbourne, VIC, Australia). Titles and abstracts were then screened for eligibility against the inclusion criteria by one reviewer (J.H.). Potentially eligible studies were obtained as full‐text articles for further screening against the inclusion criteria. A second researcher (KTM) screened 10% of the articles at the title and abstract stage and again at the full‐text stage to ensure consistency. Any study selection disagreements were resolved by discussion and consensus of both reviewers.

### 2.4 Data extraction and presentation

Data were extracted by one reviewer and charted using a piloted data charting form. This form was jointly developed by all reviewers (J.H., J.C.D., N.C. and R.E.B.W.), using Microsoft Access (Microsoft, Redmond, WA, USA), and created with reference to The Template for Intervention Description and Replication (TIDieR) checklist and guide.^16^ The variables extracted include article information (paper title, authors, and year), study information (number of participants, number of wounds, type of wounds, dressings used), collection method data (collection setting, method(s) used, volume (ml) and total protein concentration (mg/ml) of wound fluid collected, duration and frequency of collection, etc.), sample processing characteristics (processing requirements and storage conditions) and analysis methods (number of unique proteins identified and assay methods used). The study period was also calculated from the charted data, as the time between the first and last sample collection.

The data from these variables were then organised into groups based on their key characteristics. We subsequently produced 10 wound type, six collection method and 12 assay method umbrella groupings to summarise the data (Tables S1–S3). All subsequent data analysis was carried out using these groupings.

The findings were then mapped and visualised using RStudio (RStudio, Boston, MA, USA) and a descriptive numerical summary of the data given as recommended by Arksey and O'Malley.^17^ Furthermore, a narrative summary outlines the significance of the findings as well as the impact that they may have on future studies in this area.^18^

## 3 RESULTS

**FIGURE 1:**
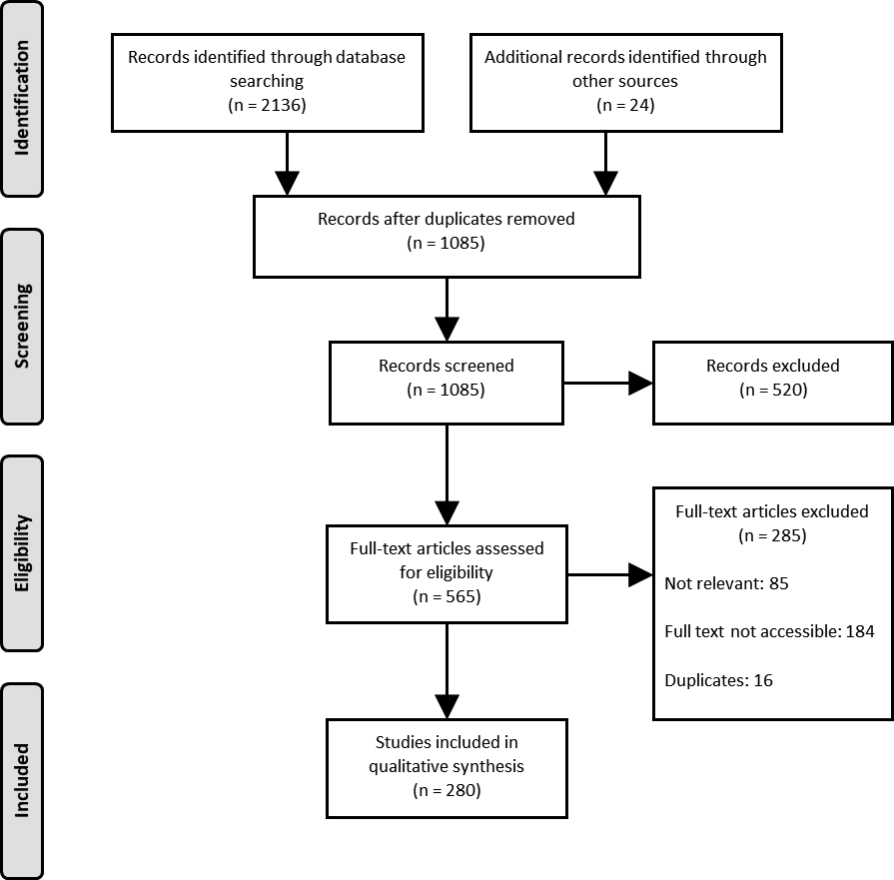
PRISMA flow diagram outlining how sources of evidence were selected

Duplicates (1075) were removed from the 2160 potentially eligible articles, leaving 1085 records for initial screening by titles and abstracts. After excluding a further 520 records, we screened the full text of 565 articles, with 280 included and subjected to data extraction (Figure 1).^8^, ^19^, ^20^, ^21^, ^22^, ^23^, ^24^, ^25^, ^26^, ^27^, ^28^, ^29^, ^30^, ^31^, ^32^, ^33^, ^34^, ^35^, ^36^, ^37^, ^38^, ^39^, ^40^, ^41^, ^42^, ^43^, ^44^, ^45^, ^46^, ^47^, ^48^, ^49^, ^50^, ^51^, ^52^, ^53^, ^54^, ^55^, ^56^, ^57^, ^58^, ^59^, ^60^, ^61^, ^62^, ^63^, ^64^, ^65^, ^66^, ^67^, ^68^, ^69^, ^70^, ^71^, ^72^, ^73^, ^74^, ^75^, ^76^, ^77^, ^78^, ^79^, ^80^, ^81^, ^82^, ^83^, ^84^, ^85^, ^86^, ^87^, ^88^, ^89^, ^90^, ^91^, ^92^, ^93^, ^94^, ^95^, ^96^, ^97^, ^98^, ^99^, ^100^, ^101^, ^102^, ^103^, ^104^, ^105^, ^106^, ^107^, ^108^, ^109^, ^110^, ^111^, ^112^, ^113^, ^114^, ^115^, ^116^, ^117^, ^118^, ^119^, ^120^, ^121^, ^122^, ^123^, ^124^, ^125^, ^126^, ^127^, ^128^, ^129^, ^130^, ^131^, ^132^, ^133^, ^134^, ^135^, ^136^, ^137^, ^138^, ^139^, ^140^, ^141^, ^142^, ^143^, ^144^, ^145^, ^146^, ^147^, ^148^, ^149^, ^150^, ^151^, ^152^, ^153^, ^154^, ^155^, ^156^, ^157^, ^158^, ^159^, ^160^, ^161^, ^162^, ^163^, ^164^, ^165^, ^166^, ^167^, ^168^, ^169^, ^170^, ^171^, ^172^, ^173^, ^174^, ^175^, ^176^, ^177^, ^178^, ^179^, ^180^, ^181^, ^182^, ^183^, ^184^, ^185^, ^186^, ^187^, ^188^, ^189^, ^190^, ^191^, ^192^, ^193^, ^194^, ^195^, ^196^, ^197^, ^198^, ^199^, ^200^, ^201^, ^202^, ^203^, ^204^, ^205^, ^206^, ^207^, ^208^, ^209^, ^210^, ^211^, ^212^, ^213^, ^214^, ^215^, ^216^, ^217^, ^218^, ^219^, ^220^, ^221^, ^222^, ^223^, ^224^, ^225^, ^226^, ^227^, ^228^, ^229^, ^230^, ^231^, ^232^, ^233^, ^234^, ^235^, ^236^, ^237^, ^238^, ^239^, ^240^, ^241^, ^242^, ^243^, ^244^, ^245^, ^246^, ^247^, ^248^, ^249^, ^250^, ^251^, ^252^, ^253^, ^254^, ^255^, ^256^, ^257^, ^258^, ^259^, ^260^, ^261^, ^262^, ^263^, ^264^, ^265^, ^266^, ^267^, ^268^, ^269^, ^270^, ^271^, ^272^, ^273^, ^274^, ^275^, ^276^, ^277^, ^278^, ^279^, ^280^, ^281^, ^282^, ^283^, ^284^, ^285^, ^286^, ^287^, ^288^, ^289^, ^290^, ^291^, ^292^, ^293^, ^294^, ^295^, ^296^, ^297^ All data relevant for answering the questions set in the scoping review protocol were extracted and are presented in the supplementary material (Table S4).

### 3.1 Overall study characteristics

The key characteristics of the included studies are summarised in Table 1. Of the 280 included studies, 203 (73%) were conducted in the last 20 years and only eight were published in 1990 or before. Hospitals were the most commonly reported setting for sample collection, although 91 studies did not report where collection took place. Of the 270 studies that reported sample size, over half had 20 participants or fewer and only five studies reported recruitment of 100 participants or more. There was large variety in the wound types studied, with a total of 409 wound groups reported across all 280 studies. There were eight instances where the type of wound was not reported, leaving 401 wound groups which could be identified. The most commonly studied groups were surgical wounds and venous leg ulcers, making up 32% (130/401) and 24% (96/401) of the identified wound groups, respectively.

**TABLE 1:** Summary of the key characteristics of all included studies

Study characteristics (*N* = 280)	*n*
Publication year	
1990 and earlier	8
1991–1995	24
1996–2000	45
2001–2005	34
2006–2010	50
2011–2015	67
2016–2020	52
Total	280
Setting	
Burn centre	10
Community clinic	14
Hospital	115
Medical centre	32
Nursing facility	6
Other settings^a^	3
Unknown setting	91
Wound healing centre	16
Total	287^b^
Number of participants	
1–20	139
21–40	92
41–60	24
61–80	6
81–100	4
Over 100	5
Unknown	10
Total	280
Wound group(s) studied	
Amputation or traumatic wound	15
Arterial ulcer	5
Artificial wound	13
Burn wound	34
Dental wound	11
Foot ulcer	37
Mixed vessel disease ulcer	9
Other wounds^c^	17
Pressure ulcer	34
Surgical wound	130
Unknown wound	8
Venous leg ulcer	96
Total	409^d^

^a^ ‘Other settings’ were defined as those that could not be classed in any of the other setting groups.

^b^ Some studies utilised multiple settings for collection. Therefore, the total number of settings (*n* = 287) exceeds the total number of studies (*n* = 280).

^c^ ‘Other wounds’ were defined as those that could not be classed in any of the other wound groups.

^d^ Some studies investigated multiple wound groups. Therefore, the total number of wound groups studied (*n* = 409) exceeds the total number of studies (*n* = 280).

### 3.2 Review questions

#### 3.2.1 Which sample collection methods have been used to collect wound fluid for the analysis of protein expression or activity?

**FIGURE 2:**
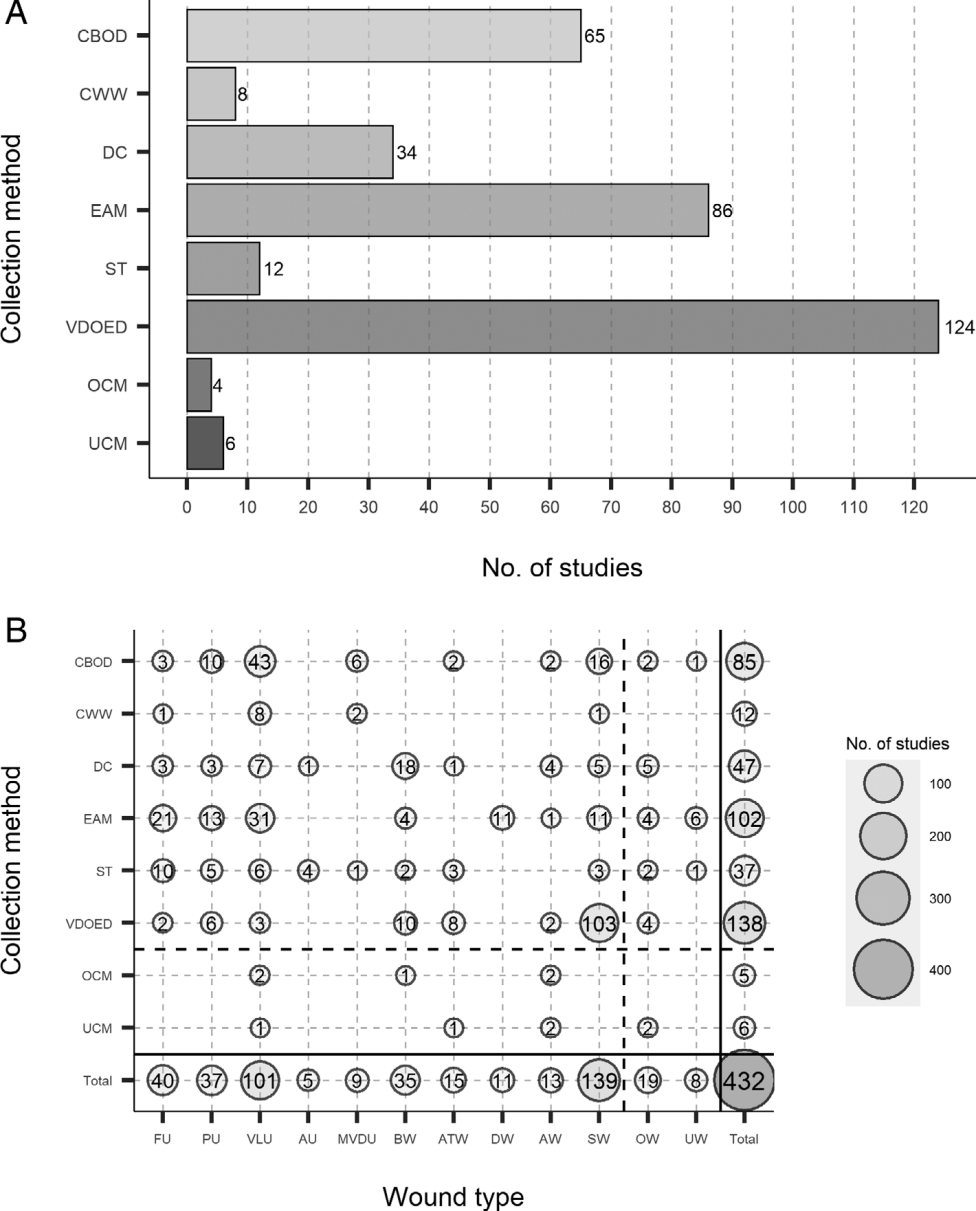
The number of studies that utilised each collection method for all included studies and all investigated wound groups. Counts were carried out for (A) the number of studies, which utilised each collection method and (B) the number and type of investigated wound groups, which utilised each collection method. Collection methods and wound types were grouped into classes as mentioned previously (Tables S1 and S2) based on description similarities. The total number of times a collection method was used in combination with all different wound groups was calculated, as well as the total number of times a wound group was used in combination with all different collection methods. As some of these studies used multiple collection methods and multiple wound groups, the totals for (A) (*n* = 339) and (B) (*n* = 432) may exceed the total number of studies (*n* = 280). AW, artificial wound; AU, arterial ulcer; ATW, amputation or traumatic wound; BW, burn wound; CBOD, collection beneath an occlusive dressing; CWW, collection of wound washout; DW, dental wound; DC, direct collection; EAM, extraction from absorbent materials; FU, foot ulcer; MVDU, mixed vessel disease ulcer; OW, other wounds; OCM, other collection methods; PU, pressure ulcer; ST, swab technique; SW, surgical wound; UW, unknown wound; UCM, unknown collection method; VDOED, vacuum, drainage or other external device; VLU, venous leg ulcer

Six distinct types of collection were identified (Figure 2A). Four studies used methods that could not be categorised into any of the six groups^19^, ^20^, ^21^, ^22^ and a further six studies did not report the collection method used for a specific wound.^23^, ^24^, ^25^, ^26^, ^27^, ^28^

Vacuum, drainage, or other external devices were used in 124 of the 280 (44%) studies and thus were the most frequently used methods of sample collection^22^, ^23^, ^24^, ^25^, ^26^, ^27^, ^29^, ^30^, ^31^, ^32^, ^33^, ^34^, ^35^, ^36^, ^37^, ^38^, ^39^, ^40^, ^41^, ^42^, ^43^, ^44^, ^45^, ^46^, ^47^, ^48^, ^49^, ^50^, ^51^, ^52^, ^53^, ^54^, ^55^, ^56^, ^57^, ^58^, ^59^, ^60^, ^61^, ^62^, ^63^, ^64^, ^65^, ^66^, ^67^, ^68^, ^69^, ^70^, ^71^, ^72^, ^73^, ^74^, ^75^, ^76^, ^77^, ^78^, ^79^, ^80^, ^81^, ^82^, ^83^, ^84^, ^85^, ^86^, ^87^, ^88^, ^89^, ^90^, ^91^, ^92^, ^93^, ^94^, ^95^, ^96^, ^97^, ^98^, ^99^, ^100^, ^101^, ^102^, ^103^, ^104^, ^105^, ^106^, ^107^, ^108^, ^109^, ^110^, ^111^, ^112^, ^113^, ^114^, ^115^, ^116^, ^117^, ^118^, ^119^, ^120^, ^121^, ^122^, ^123^, ^124^, ^125^, ^126^, ^127^, ^128^, ^129^, ^130^, ^131^, ^132^, ^133^, ^134^, ^135^, ^136^, ^137^, ^138^, ^139^, ^140^, ^141^, ^142^, ^143^, ^144^, ^145^, ^146^; the vast majority of sample collections using this method (75%, 103/138), were from surgical wounds (Figure 2B). Three other frequently used methods were extraction from absorbent material (31%, 86/280),^8^, ^20^, ^37^, ^81^, ^89^, ^136^, ^138^, ^144^, ^146^, ^147^, ^148^, ^149^, ^150^, ^151^, ^152^, ^153^, ^154^, ^155^, ^156^, ^157^, ^158^, ^159^, ^160^, ^161^, ^162^, ^163^, ^164^, ^165^, ^166^, ^167^, ^168^, ^169^, ^170^, ^171^, ^172^, ^173^, ^174^, ^175^, ^176^, ^177^, ^178^, ^179^, ^180^, ^181^, ^182^, ^183^, ^184^, ^185^, ^186^, ^187^, ^188^, ^189^, ^190^, ^191^, ^192^, ^193^, ^194^, ^195^, ^196^, ^197^, ^198^, ^199^, ^200^, ^201^, ^202^, ^203^, ^204^, ^205^, ^206^, ^207^, ^208^, ^209^, ^210^, ^211^, ^212^, ^213^, ^214^, ^215^, ^216^, ^217^, ^218^, ^219^, ^220^, ^221^, ^222^, ^223^ collection beneath occlusive dressing (23%, 65/280)^22^, ^24^, ^27^, ^29^, ^33^, ^36^, ^41^, ^43^, ^53^, ^62^, ^64^, ^66^, ^67^, ^68^, ^72^, ^77^, ^80^, ^86^, ^88^, ^95^, ^107^, ^111^, ^114^, ^116^, ^129^, ^145^, ^146^, ^169^, ^174^, ^224^, ^225^, ^226^, ^227^, ^228^, ^229^, ^230^, ^231^, ^232^, ^233^, ^234^, ^235^, ^236^, ^237^, ^238^, ^239^, ^240^, ^241^, ^242^, ^243^, ^244^, ^245^, ^246^, ^247^, ^248^, ^249^, ^250^, ^251^, ^252^, ^253^, ^254^, ^255^, ^256^, ^257^, ^258^, ^259^ and direct collection (12%, 34/280).^23^, ^26^, ^42^, ^53^, ^99^, ^113^, ^198^, ^225^, ^242^, ^245^, ^248^, ^251^, ^260^, ^261^, ^262^, ^263^, ^264^, ^265^, ^266^, ^267^, ^268^, ^269^, ^270^, ^271^, ^272^, ^273^, ^274^, ^275^, ^276^, ^277^, ^278^, ^279^, ^280^, ^281^ The wound groups most frequently sampled using extraction from absorbent material or collection beneath occlusive dressing were ulcers: either pressure, foot, or most commonly, venous leg ulcers, whereas direct collection was more often used to sample burns. The two other defined methods, swab technique (4%, 12/280)^129^, ^214^, ^227^, ^282^, ^283^, ^284^, ^285^, ^286^, ^287^, ^288^, ^289^, ^290^ and collection of wound washout (3%, 8/280)^273^, ^291^, ^292^, ^293^, ^294^, ^295^, ^296^, ^297^ were used in only a small proportion of all included studies.

##### What study designs and sampling regiments have been used (number of samples and frequency of wound sampling)?

Tables 2 and 3 summarise the sampling regimens of all investigated wound groups (*n* = 409) from the included studies. Of these wound groups, 17 did not record the number of samples collected per wound and 26 did not clearly report the time between each sample collection, leaving a total of 392 and 383 investigated wound groups with reported sampling details for the data presented in Tables 2 and 3, respectively. Most of the studies were cross‐sectional and took only one sample per wound. Where studies took multiple samples from each wound over time, the number of samples collected was usually small, typically 2–3. Collecting over 10 samples per wound was uncommon and only occurred in nine of the investigated wound groups. The time between each collection, for longitudinal designs, was typically 1–3 days accounting for 21% (82/383) of the wound groups with reported sampling details. Consequently, most longitudinal studies had short study durations and small sample sizes.

**TABLE 2:** Number of samples collected per wound, for each wound type in all included studies, grouped by collection method

No. of samples collected (per wound)	Collection beneath occlusive dressing	Collection of wound washout	Direct collection	Extraction from absorbent materials	Swab technique	Vacuum, drainage or other external device	Other collection methods	Unknown collection method	All studies^a^
	% (*n* ^b^)	% (*n*)	% (*n*)	% (*n*)	% (*n*)	% (*n*)	% (*n*)	% (*n*)	% (*n*)
1	54 (46)	58 (7)	83 (39)	49 (50)	78 (29)	41 (57)	60 (3)	83 (5)	53 (215)
2–3	18 (15)	33 (4)	2 (1)	10 (10)	19 (7)	16 (22)	20 (1)	0 (0)	14 (59)
4–5	6 (5)	0 (0)	11 (5)	15 (15)	0 (0)	9 (13)	0 (0)	0 (0)	9 (38)
6–10	6 (5)	0 (0)	0 (0)	11 (11)	0 (0)	8 (11)	0 (0)	0 (0)	7 (27)
>10	0 (0)	0 (0)	0 (0)	5 (5)	3 (1)	1 (2)	20 (1)	0 (0)	2 (9)
Varied^c^	5 (4)	8 (1)	2 (1)	8 (8)	0 (0)	17 (24)	0 (0)	17 (1)	11 (44)
Unknown	12 (10)	0 (0)	2 (1)	3 (3)	0 (0)	7 (9)	0 (0)	0 (0)	4 (17)
Total	100 (85)	100 (12)	100 (47)	100 (102)	100 (37)	100 (138)	100 (5)	100 (6)	100 (409^d^)

^a^ ‘All studies’ refers to the number of wound groups studied regardless of collection method used.

^b^ *n* = number of wound groups studied.

^c^ ‘Varied’ refers to the collection of different numbers of samples for different wounds in a group.

^d^ Some studies used multiple collection methods. Therefore, the sum total of wound groups studied for all collection methods (*n* = 432) is higher than the total number of wound groups for all studies (*n* = 409), as some wound groups have been counted multiple times.

**TABLE 3:** Time taken between each sample collection, for each wound type in all included studies, grouped by collection method

Time between sample collections	Collection beneath occlusive dressing	Collection of wound washout	Direct collection	Extraction from absorbent materials	Swab technique	Vacuum, drainage or other external device	Other collection methods	Unknown collection method	All studies^a^
	% (*n* ^b^)	% (*n*)	% (*n*)	% (*n*)	% (*n*)	% (*n*)	% (*n*)	% (*n*)	% (*n*)
<1 day	0 (0)	0 (0)	0 (0)	0 (0)	0 (0)	9 (12)	0 (0)	0 (0)	3 (12)
1–3 days	14 (12)	0 (0)	4 (2)	11 (11)	0 (0)	41 (57)	0 (0)	17 (1)	20 (82)
4–6 days	0 (0)	0 (0)	0 (0)	1 (1)	0 (0)	1 (1)	0 (0)	0 (0)	0 (2)
1–2 weeks	6 (5)	33 (4)	9 (4)	14 (14)	5 (2)	1 (2)	0 (0)	0 (0)	8 (31)
2–4 weeks	13 (11)	0 (0)	2 (1)	2 (2)	0 (0)	0 (0)	0 (0)	0 (0)	3 (14)
>1 month	5 (4)	0 (0)	0 (0)	3 (3)	0 (0)	0 (0)	0 (0)	0 (0)	2 (7)
Varied^c^	1 (1)	0 (0)	0 (0)	9 (9)	0 (0)	2 (3)	20 (1)	0 (0)	3 (13)
All at once^d^	0 (0)	0 (0)	0 (0)	6 (6)	0 (0)	0 (0)	20 (1)	0 (0)	2 (7)
N/A^e^	54 (46)	58 (7)	83 (39)	49 (50)	78 (29)	41 (57)	60 (3)	83 (5)	53 (215)
Unknown	7 (6)	8 (1)	2 (1)	6 (6)	16 (6)	4 (6)	0 (0)	0 (0)	6 (26)
Total	100 (85)	100 (12)	100 (47)	100 (102)	100 (37)	100 (138)	100 (5)	100 (6)	100 (409^f^)

^a^ ‘All studies’ refers to the number of wound groups studied regardless of collection method used.

^b^ *n* = number of wound groups studied.

^c^ If there was not a specific time between each collection which made up the majority, then the time was charted as ‘Varied’.

^d^ ‘All at once’ refers to all instances where multiple samples were collected at the same time.

^e^ ‘N/A' refers to all instances where only one sample was collected.

^f^ Some studies used multiple collection methods. Therefore, the sum total of studied wound groups for all collection methods (*n* = 432) is higher than the total number of wound groups for all studies (*n* = 409), as some wound groups have been counted multiple times.

##### How are wound fluid samples processed and stored before proteomic analysis?

Sixteen studies did not report how samples were processed and four did not report storage conditions (Tables 4 and 5) leaving a total of 264 and 276 studies with reported sample conditions, respectively. The majority of studies which reported these conditions processed samples immediately (71%, 188/264) and used ultra‐low freezer temperatures of −70°C or −80°C for sample storage (68%, 189/276). A further 16% (43/276) stored samples at −20°C and only four studies kept samples at over 0°C. Finally, only 14% (38/276) of studies processed and analysed samples immediately with no storage of samples before the initial analysis.

**TABLE 4:** The sample processing characteristics of all studies, grouped by collection method

Immediate processing?	Collection beneath occlusive dressing	Collection of wound washout	Direct collection	Extraction from absorbent materials	Swab technique	Vacuum, drainage or other external device	Other collection methods	Unknown collection method	All studies^a^
	% (*n* ^b^)	% (*n*)	% (*n*)	% (*n*)	% (*n*)	% (*n*)	% (*n*)	% (*n*)	% (*n*)
No	14 (9)	12 (1)	26 (9)	37 (32)	42 (5)	17 (21)	25 (1)	0 (0)	26 (73)
Yes	83 (54)	88 (7)	56 (19)	58 (50)	42 (5)	76 (94)	50 (2)	100 (6)	67 (188)
If possible^c^	2 (1)	0 (0)	6 (2)	1 (1)	0 (0)	1 (1)	0 (0)	0 (0)	1 (3)
Unknown	2 (1)	0 (0)	12 (4)	3 (3)	17 (2)	6 (8)	25 (1)	0 (0)	6 (16)
Total	100 (65)	100 (8)	100 (34)	100 (86)	100 (12)	100 (124)	100 (4)	100 (6)	100 (280^d^)

^a^ ‘All studies’ refers to the number of wound groups studied regardless of collection method used.

^b^ *n* = Number of studies.

^c^ ‘If possible’ refers to studies which attempted immediate processing but where it may not have been possible for all samples.

^d^ Some studies used multiple collection methods. Therefore, the sum total of studies for all collection methods (*n* = 339) is higher than the total number of studies (*n* = 280), as some studies have been counted multiple times.

**TABLE 5:** The sample storage conditions of all studies, grouped by collection method

Sample storage conditions	Collection beneath occlusive dressing	Collection of wound washout	Direct collection	Extraction from absorbent materials	Swab technique	Vacuum, drainage or other external device	Other collection methods	Unknown collection method	All studies^a^
	% (*n* ^b^)	% (*n*)	% (*n*)	% (*n*)	% (*n*)	% (*n*)	% (*n*)	% (*n*)	% (*n*)
−80°C	43 (28)	75 (6)	24 (8)	59 (51)	42 (5)	44 (54)	25 (1)	33 (2)	48 (134)
−70°C	26 (17)	0 (0)	32 (11)	13 (11)	8 (1)	23 (28)	25 (1)	33 (2)	20 (55)
−20°C	20 (13)	0 (0)	21 (7)	15 (13)	17 (2)	16 (20)	25 (1)	17 (1)	15 (43)
4°C	2 (1)	0 (0)	0 (0)	1 (1)	8 (1)	1 (1)	0 (0)	0 (0)	1 (3)
16°C	0 (0)	0 (0)	3 (1)	0 (0)	0 (0)	0 (0)	0 (0)	0 (0)	0 (1)
Other^c^	0 (0)	0 (0)	0 (0)	0 (0)	8 (1)	1 (1)	0 (0)	0 (0)	1 (2)
N/A^d^	8 (5)	12 (1)	18 (6)	12 (10)	17 (2)	15 (19)	25 (1)	17 (1)	14 (38)
Unknown	2 (1)	12 (1)	3 (1)	0 (0)	0 (0)	1 (1)	0 (0)	0 (0)	1 (4)
Total	100 (65)	100 (8)	100 (34)	100 (86)	100 (12)	100 (124)	100 (4)	100 (6)	100 (280^e^)

^a^ ‘All studies’ refers to the number of wound groups studied regardless of collection method used.

^b^ *n* = Number of studies.

^c^ ‘Other’ sample storage conditions were those that could not be defined by any of the other charted categories.

^d^ ‘N/A’ refers to studies which did not store their samples before processing or initial analysis.

^e^ Some studies used multiple collection methods. Therefore, the sum total of studies for all collection methods (*n* = 339) is higher than the total number of studies (*n* = 280), as some studies have been counted multiple times.

##### Which proteomic analysis methods are used on the collected samples and does this influence the method of collection used?

**FIGURE 3:**
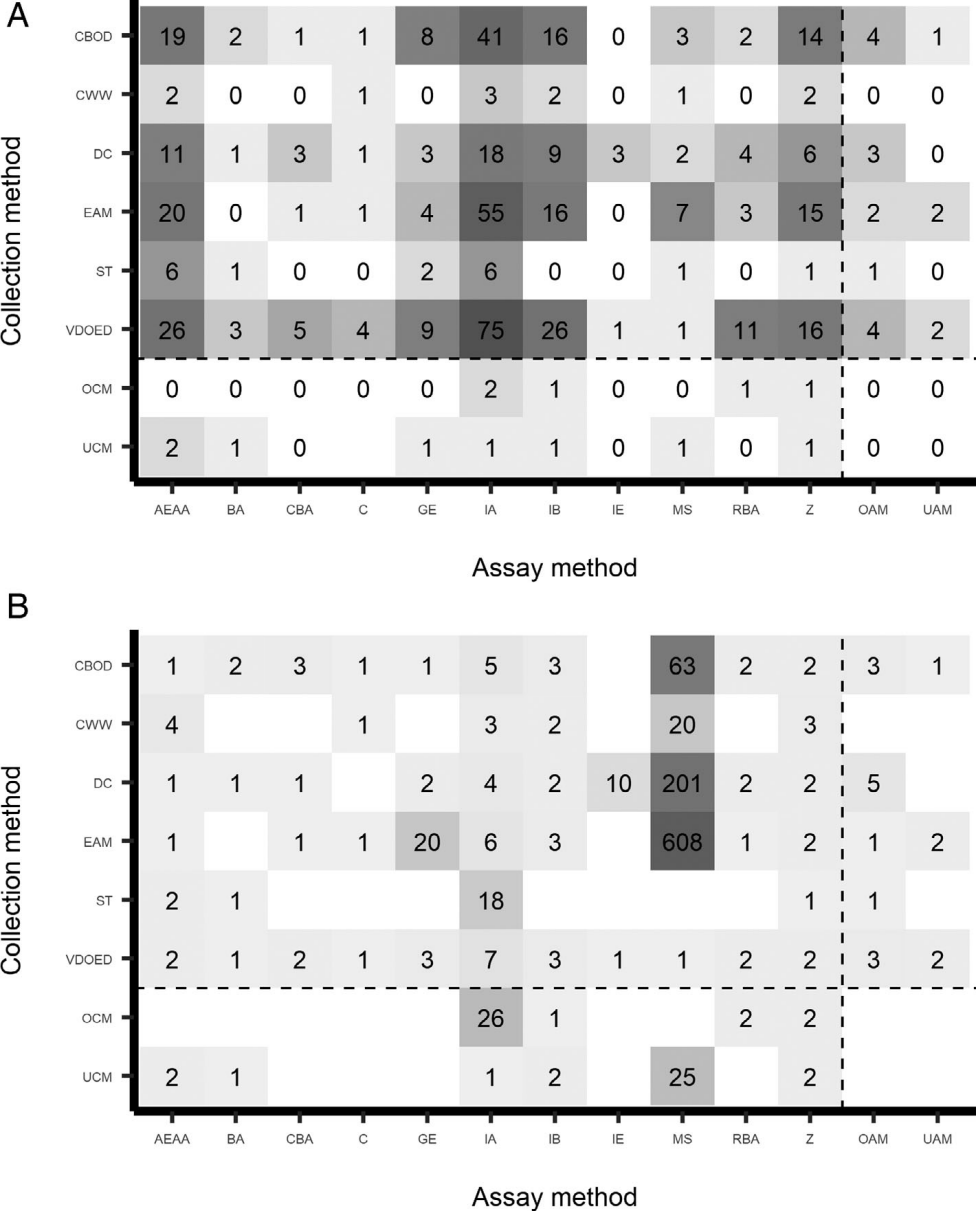
The number of studies that utilised each combination of assay and collection methods and the average number of unique proteins identified by each of these combinations. All studies from which data was extracted were included here (*n* = 280). (A) Counts were carried out for the number of studies which utilised each collection—assay combination. Some studies may have used multiple collection and/or assay methods. Therefore, the total for all combinations used (*n* = 561) may exceed the total number of studies (*n* = 280). (B) The average number of unique proteins identified in studies which used each collection—assay combination were calculated. Mean averages for each combination were rounded to the nearest integer value. Combinations which were not utilised by any studies, or where no unique protein identifications were reported, are denoted by a blank space. AEAA, absorbance or enzyme activity assay; BA, bioassay, CBA, cell‐based assay; C, chromatography; CBOD, collection beneath an occlusive dressing; CWW, collection of wound washout; DC, direct collection; EAM, extraction from absorbent materials; GE, gel electrophoresis; IA, immunoassay; IB, immunoblot; IE, immunoelectrophoresis; MS, mass spectrometry; OAM, other assay methods; OCM, other collection methods; RBA, radiation‐based assay; ST, swab technique; UAM, unknown assay method; UCM, unknown collection method; VDOED, vacuum, drainage or other external device; Z, zymography

Immunoassays were the most frequently used method of analysis for all collection methods (Figure 3A). Other commonly used assay methods included absorbance or enzyme activity assays, immunoblot and zymography. Some of the assay methods were only utilised by studies using specific collection methods. For example, immunoelectrophoresis was only utilised for sample analysis when either direct collection or vacuum, drainage or other external devices were used to obtain samples. The majority of studies used collection – assay combinations which, on average, identified relatively few unique proteins (Figure 3B). Whilst techniques such as mass spectrometry, which when used in an untargeted workflow aim for complete proteome coverage, were utilised by relatively few studies.^298^ When it was used, mass spectrometry was most frequently combined with collection via extraction from absorbent materials and the average number of unique protein identifications was highest when using this collection – assay approach. The number of unique proteins identified in each study did not correlate with either volume (ml) or total protein concentration (mg/ml) of wound fluid collected (Figures S1 and S2). Although these details of collection were only recorded for 100 and 41 of the 280 included studies, respectively.

## 4 DISCUSSION

### 4.1 Summary and implication of findings

We included 280 eligible studies, which collected wound fluid samples from human participants. Mapping of the data has highlighted the considerable heterogeneity present in wound fluid sampling, with at least six methodologically distinct collection methods being used. There was further variation within each of these groups with the details of collection differing between studies. This heterogeneity of activity and reporting impacts on confident comparisons between studies: various sampling methods are likely to influence the recovery of individual analytes, especially if processing and storage methods were also disparate.^129^, ^299^ Comparisons may only be possible between studies using similar sampling, processing and storage methods and could limit the value of the body of evidence.^14^

Some sample collection methods are more commonly used than others. Rationales to support choice of collection method are not clear but may be primarily driven by wound type. The most commonly used collection methods – vacuum, drainage or other external devices – are primarily used for collection of wound fluid from surgical wounds (the most commonly chosen wound type). Drainage of fluid from surgical wounds is already common clinical practise,^300^ where large volumes of exudate can be produced thus providing readily available samples for non‐invasive collection.^14^ Exudate volume may also influence the collection method used for other wound types. For example, direct collection of wound fluid will typically require a larger volume than other methods, which may explain its use in sampling from burns or venous leg ulcers, which generally produce higher exudate volumes than some other non‐surgical wound types.^301^

Many of the included studies utilised a cross‐sectional design, with samples taken at a single time point.^302^ Studies designed in this manner are usually easy and inexpensive to set up but are not an appropriate design for assessing associations between potentially prognostic factors (such as biomarkers) and clinical outcomes,^303^ nor wound healing research more broadly. Timing of sample collection is crucial, particularly as wounds will be in one of several possible healing phases,^304^ with protein concentrations fluctuating accordingly.^222^ Taking samples during only one of these phases will thus miss the complexity inherent in the healing process and may provide misleading results. Longitudinal follow‐up is also crucial to enable meaningful clinical endpoints such as complete wound healing to be assessed.

Although some of the included studies^160^, ^164^, ^231^, ^237^, ^290^ did embed sample collection in a longitudinal design, the number of samples collected was typically low and the study periods short. This means although the evidence base we have identified is large, it does not identify an obvious wound fluid sampling approach for longer studies. Therefore, further work could focus on exploring optimal wound fluid sampling methods to be used in longitudinal studies in terms of being valid and operationally feasible in clinical settings.

For untargeted proteomics and biomarker discovery studies, techniques that allow for identification and measurement of large protein numbers simultaneously, such as mass spectrometry or multiplex immunoassays, are often favoured.^305^ The relatively low number of studies which utilised such techniques therefore suggests that these study types are in the minority for wound fluid investigations. As measured wound fluid characteristics (wound fluid volume and total protein concentration) did not affect the number of proteins identified by a study, the choice of collection method may be due to other factors, such as the type of wound sampled.

Future work in this area should focus on identification of valid sampling approaches for wound proteomic studies ensuring that research waste is minimised by learning from studies that have already been conducted and reported. Whilst the 280 included studies in this scoping review represent large investment of time and other resources, the heterogeneity and size and scope of the evidence limits the value of the existing evidence for guiding sampling approaches for future work, thus perpetuating these issues. To support the conduct of rigorous biomarker identification and validation studies, sampling and analytical processes need to be proven accurate, reproducible and feasible for use in larger cohorts.^306^ There is also a lack of recognised guidelines for the collection of wound fluid, using the included methods. Therefore, the development of a set of sampling standards may benefit further work in this area. Future studies that aim to link wound fluid sampling analysis to clinical outcomes should also draw on epidemiological as well as biological methodologies to ensure application of rigorous prognostic research methodology.^307^

### 4.2 Strengths and limitations

This review is the first of its kind to summarise the methods used to sample, process, store and analyse fluid from human wounds. We followed a systematic, pre‐determined approach to the review, documented in a protocol, and ensured consistency by, for example, developing and piloting a data extraction form to best capture all data relevant for addressing the review questions.

Some deviations were made from the original protocol on commencement of data collection, extraction, and charting but none of these were considered to introduce bias into the process. Firstly, it was decided that related evidence that has supported development or use of the method would not be included, as this was outside the scope of the review; however, the data may be useful for research in this area. Furthermore, animal studies that used wound fluid for proteomics research were omitted, as data from these were deemed not relevant in answering the questions posed. As stated in the protocol, all articles not written in English and those not accessible as full texts were removed during the screening stage. These articles may include information relevant to this scoping review but were removed due to time and resource constraints.

Additionally, screening was carried out by one reviewer which increases the risk of missing relevant studies^308^, ^309^ and the possibility of selection bias.^310^ To minimise the likelihood of this, a second reviewer screened 10% of the articles at the abstract and full‐text stages. Disagreements were uncommon, but where these occurred consensus between both reviewers was required before moving on and these decisions influenced the rest of the screening process. Furthermore, a single screening system was time intensive. To streamline the process in future scoping reviews, it may be preferable to use screening software, which employs a text‐mining tool to prioritise potentially eligible studies.^311^, ^312^

As the amount of data extracted was relatively large, groupings were created for some of the charted variables. Groups were created to clarify and summarise the key elements and allow clear visualisation of the extracted data, although grouping of some of these variables may be viewed as subjective. To minimise the risk of bias, all groupings were created by one researcher before consultation and acceptance by two other researchers. However, grouping the data in this way masks any variations within a group that may have been of interest.

## 5 CONCLUSIONS

This scoping review successfully mapped and numerically summarised the methods used for collection of wound fluid samples for protein analysis. This demonstrated that a large variety of collection methods have been employed, with some methods being used far more frequently than others. The use of specific methods may be dictated by the type of wound under study and the clinical characteristics of that wound. The majority of studies used small sample sizes and had short study periods, with many opting for a cross‐sectional design. Many proteomics studies, such as those for the identification and validation of biomarkers, require larger longitudinal designs to yield useful results. This review therefore highlights the requirement for progression in wound healing research to focus on larger cohort sizes over extended study periods, to acquire robust data for wound proteomics investigations. The data presented herein should reduce research waste by allowing researchers to learn from past studies and to address knowledge gaps in our existing understanding of complex wounds. Future work should aim to rigorously assess sampling methods and associated analytical techniques for use in large prognosis studies, with the possibility to create a set of sampling standards to ensure consistency between studies.

## CONFLICT OF INTEREST

The authors declare no conflict of interest.

## Supporting information


Appendix S1. Supporting Information

## Data Availability

The data that supports the findings of this study are available in the supplementary material of this article
