# Supplementary material for: Wound fluid sampling methods for proteomic studies: A scoping review
Source: Wound Repair Regen. 2022 Apr 5;30(3):317–33. doi: 10.1111/wrr.13009 (PMC9322564; doi:10.1111/wrr.13009)
Supplement: Supplementary file 1 — Appendix S1. Supporting Information [file WRR-30-317-s001.docx]

**Appendix S1. Preferred Reporting Items for Systematic reviews and Meta-Analyses extension for Scoping Reviews (PRISMA-ScR) Checklist**

| **SECTION** | **ITEM** | **PRISMA-ScR CHECKLIST ITEM** | **REPORTED ON PAGE #** |
| --- | --- | --- | --- |
| **TITLE** | | | |
| Title | 1 | Identify the report as a scoping review. | 1 |
| **ABSTRACT** | | | |
| Structured summary | 2 | Provide a structured summary that includes (as applicable): background, objectives, eligibility criteria, sources of evidence, charting methods, results, and conclusions that relate to the review questions and objectives. | 2 |
| **INTRODUCTION** | | | |
| Rationale | 3 | Describe the rationale for the review in the context of what is already known. Explain why the review questions/objectives lend themselves to a scoping review approach. | 3 |
| Objectives | 4 | Provide an explicit statement of the questions and objectives being addressed with reference to their key elements (e.g., population or participants, concepts, and context) or other relevant key elements used to conceptualize the review questions and/or objectives. | 4 |
| **METHODS** | | | |
| Protocol and registration | 5 | Indicate whether a review protocol exists; state if and where it can be accessed (e.g., a Web address); and if available, provide registration information, including the registration number. | 4 |
| Eligibility criteria | 6 | Specify characteristics of the sources of evidence used as eligibility criteria (e.g., years considered, language, and publication status), and provide a rationale. | 4-5 |
| Information sources* | 7 | Describe all information sources in the search (e.g., databases with dates of coverage and contact with authors to identify additional sources), as well as the date the most recent search was executed. | 5 |
| Search | 8 | Present the full electronic search strategy for at least 1 database, including any limits used, such that it could be repeated. | 5, Appendix S1 |
| Selection of sources of evidence† | 9 | State the process for selecting sources of evidence (i.e., screening and eligibility) included in the scoping review. | 5 |
| Data charting process‡ | 10 | Describe the methods of charting data from the included sources of evidence (e.g., calibrated forms or forms that have been tested by the team before their use, and whether data charting was done independently or in duplicate) and any processes for obtaining and confirming data from investigators. | 6 |
| Data items | 11 | List and define all variables for which data were sought and any assumptions and simplifications made. | 6, Table S1-S3 |
| Critical appraisal of individual sources of evidence§ | 12 | If done, provide a rationale for conducting a critical appraisal of included sources of evidence; describe the methods used and how this information was used in any data synthesis (if appropriate). | NA |
| Synthesis of results | 13 | Describe the methods of handling and summarizing the data that were charted. | 6 |
| **RESULTS** | | | |
| Selection of sources of evidence | 14 | Give numbers of sources of evidence screened, assessed for eligibility, and included in the review, with reasons for exclusions at each stage, ideally using a flow diagram. | 7, Fig. 1 |
| Characteristics of sources of evidence | 15 | For each source of evidence, present characteristics for which data were charted and provide the citations. | 7, Table 1 |
| Critical appraisal within sources of evidence | 16 | If done, present data on critical appraisal of included sources of evidence (see item 12). | NA |
| Results of individual sources of evidence | 17 | For each included source of evidence, present the relevant data that were charted that relate to the review questions and objectives. | 7, Table S4 |
| Synthesis of results | 18 | Summarize and/or present the charting results as they relate to the review questions and objectives. | 7-9, Table 2-5, Fig. 2-5 |
| **DISCUSSION** | | | |
| Summary of evidence | 19 | Summarize the main results (including an overview of concepts, themes, and types of evidence available), link to the review questions and objectives, and consider the relevance to key groups. | 10-12 |
| Limitations | 20 | Discuss the limitations of the scoping review process. | 12-13 |
| Conclusions | 21 | Provide a general interpretation of the results with respect to the review questions and objectives, as well as potential implications and/or next steps. | 13-14 |
| **FUNDING** | | | |
| Funding | 22 | Describe sources of funding for the included sources of evidence, as well as sources of funding for the scoping review. Describe the role of the funders of the scoping review. | 14 |

JBI = Joanna Briggs Institute; PRISMA-ScR = Preferred Reporting Items for Systematic reviews and Meta-Analyses extension for Scoping Reviews.

* Where *sources of evidence* (see second footnote) are compiled from, such as bibliographic databases, social media platforms, and Web sites.

† A more inclusive/heterogeneous term used to account for the different types of evidence or data sources (e.g., quantitative and/or qualitative research, expert opinion, and policy documents) that may be eligible in a scoping review as opposed to only studies. This is not to be confused with *information sources* (see first footnote).

‡ The frameworks by Arksey and O’Malley (6) and Levac and colleagues (7) and the JBI guidance (4, 5) refer to the process of data extraction in a scoping review as data charting*.*

§ The process of systematically examining research evidence to assess its validity, results, and relevance before using it to inform a decision. This term is used for items 12 and 19 instead of "risk of bias" (which is more applicable to systematic reviews of interventions) to include and acknowledge the various sources of evidence that may be used in a scoping review (e.g., quantitative and/or qualitative research, expert opinion, and policy document).

*From:* Tricco AC, Lillie E, Zarin W, O'Brien KK, Colquhoun H, Levac D, et al. PRISMA Extension for Scoping Reviews (PRISMAScR): Checklist and Explanation. Ann Intern Med. 2018;169:467–473. [doi: 10.7326/M18-0850](http://annals.org/aim/fullarticle/2700389/prisma-extension-scoping-reviews-prisma-scr-checklist-explanation).

**Appendix S2.** Search strategy

**Ovid (MEDLINE): Open wounds**

Database(s): Ovid MEDLINE(R) and Epub Ahead of Print, In-Process & Other Non-Indexed Citations, Daily and Versions(R) 1946 to May 18, 2020

1. exp Specimen Handling/ (346114)

2. (sampl* adj technique*).tw. (9202)

3. (sampl* adj approach*).tw. (1905)

4. (sampl* adj procedure*).tw. (3568)

5. (experiment* adj technique*).tw. (6175)

6. (experiment* adj approach*).tw. (19616)

7. (experiment* adj procedure*).tw. (6577)

8. 1 or 2 or 3 or 4 or 5 or 6 or 7 (391433)

9. exp Proteins/ (6281988)

10. exp Enzymes/ (2942906)

11. exp Protease Inhibitors/ (201431)

12. exp Peptides/ (2695371)

13. (protein* adj expres*).tw. (153444)

14. (protein* adj activit*).tw. (7268)

15. (protein* adj extract**).tw. (9689)

16. protein* inhibit* expres*.tw. (37)

17. protein* inhibit* activit*.tw. (252)

18. (proteas* or proteinas*).tw. (204141)

19. (proteas* adj (expres* or activit*)).tw. (13552)

20. ((up-regulation or down-regulation or accumulat* or reduct*) adj3 (proteas* or proteinas*)).tw. (1463)

21. 9 or 10 or 11 or 12 or 13 or 14 or 15 or 16 or 17 or 18 or 19 or 20 (7657308)

22. 8 or 21 (7958857)

23. exp Leg Ulcer/ (22374)

24. exp Foot Ulcer/ (10157)

25. exp Pressure Ulcer/ (12233)

26. exp Diabetic Foot/ (8778)

27. exp Wounds, Penetrating/ (36147)

28. exp Lacerations/ (3275)

29. exp Burns/ (57342)

30. exp Wound Infection/ (46720)

31. exp Abscess/ (56082)

32. exp Pilonidal Sinus/ (1891)

33. exp "Bites and Stings"/ (21179)

34. exp Cicatrix/ (38860)

35. exp Wound Healing/ (125476)

36. exp Soft Tissue Infections/ (3557)

37. ((plantar or diabetic or heel$ or foot or feet or ischaemic or ischemic or venous or varicose or stasis or arterial or decubitus or pressure or skin or leg or mixed) adj5 (wound$ or ulcer$)).tw. (51921)

38. (bedsore* or bed sore*).tw. (697)

39. (pilonidal sinus* or pilonidal cyst*).tw. (1563)

40. (cavity wound* or sinus wound*).tw. (97)

41. (laceration* or gunshot or stab or stabbing or stabbed or bite*).tw. (58137)

42. (burn or burns or burned or scald*).tw. (64239)

43. (wound* adj5 dehisc*).tw. (5157)

44. (wound* adj5 infect*).tw. (34426)

45. (wound* adj5 disrupt*).tw. (731)

46. wound complication*.tw. (5689)

47. (malignant wound* or experimental wound* or traumatic wound*).tw. (1319)

48. (infusion site* or donor site* or wound site*).tw. (18244)

49. (skin abscess* or skin abcess*).tw. (473)

50. exp Exudates/ and Transudates/ (10425)

51. (wound* adj3 (sampl* or fluid* or exudate* or discharge or effluent*)).tw. (3253)

52. 23 or 24 or 25 or 26 or 27 or 28 or 29 or 30 or 31 or 32 or 33 or 34 or 35 or 36 or 37 or 38 or 39 or 40 or 41 or 42 or 43 or 44 or 45 or 46 or 47 or 48 or 49 or 50 (495903)

53. 51 and 52 (2478)

54. 22 and 53 (919)

55. human* volunteer*.tw. (8964)

56. volunt*.tw. (256879)

57. 55 or 56 (256879)

58. 54 and 57 (20)

59. exp animals/ not humans.sh. (4699122)

60. 54 not 59 (634)

61. 58 or 60 (634)

**Ovid (MEDLINE): Surgical wounds**

Database(s): Ovid MEDLINE(R) and Epub Ahead of Print, In-Process & Other Non-Indexed Citations, Daily and Versions(R) 1946 to May 18, 2020

1. exp Specimen Handling/ (346114)

2. (sampl* adj technique*).tw. (9202)

3. (sampl* adj approach*).tw. (1905)

4. (sampl* adj procedure*).tw. (3568)

5. (experiment* adj technique*).tw. (6175)

6. (experiment* adj approach*).tw. (19616)

7. (experiment* adj procedure*).tw. (6577)

8. 1 or 2 or 3 or 4 or 5 or 6 or 7 (391433)

9. exp Proteins/ (6281988)

10. exp Enzymes/ (2942906)

11. exp Protease Inhibitors/ (201431)

12. exp Peptides/ (2695371)

13. (protein* adj expres*).tw. (153444)

14. (protein* adj activit*).tw. (7268)

15. (protein* adj extract**).tw. (9689)

16. protein* inhibit* expres*.tw. (37)

17. protein* inhibit* activit*.tw. (252)

18. (proteas* or proteinas*).tw. (204141)

19. (proteas* adj (expres* or activit*)).tw. (13552)

20. ((up-regulation or down-regulation or accumulat* or reduct*) adj3 (proteas* or proteinas*)).tw. (1463)

21. 9 or 10 or 11 or 12 or 13 or 14 or 15 or 16 or 17 or 18 or 19 or 20 (7657308)

22. 8 or 21 (7958857)

23. exp Surgical Wound/ (815)

24. exp Surgical Wound Infection/ (36128)

25. exp Surgical Wound Dehiscence/ (7386)

26. exp Exudates/ and Transudates/ (10425)

27. exp Drainage/ (58809)

28. (surg* adj5 infect*).tw. (33569)

29. (surg* adj5 wound*).tw. (15617)

30. (surg* adj5 site*).tw. (24592)

31. (surg* adj5 incision*).tw. (11215)

32. (surg* adj5 dehisc*).tw. (1181)

33. (wound* adj5 dehisc*).tw. (5157)

34. (wound* adj5 infect*).tw. (34426)

35. (wound adj5 disrupt*).tw. (612)

36. wound complication*.tw. (5689)

37. (drain* adj3 fluid*).tw. (5006)

38. 23 or 24 or 25 or 26 or 27 or 28 or 29 or 30 or 31 or 32 or 33 or 34 or 35 or 36 or 37 (192056)

39. (wound* adj3 (sampl* or fluid* or exudate* or discharge or effluent*)).tw. (3253)

40. 22 and 38 and 39 (442)

41. (human* adj3 volunteer*).tw. (9791)

42. volunt*.tw. (256879)

43. 41 or 42 (256879)

44. 40 and 43 (10)

45. exp animals/ not humans.sh. (4699122)

46. 40 not 45 (361)

47. 44 or 46 (361)

48. limit 40 to humans (348)

**Ovid (Embase): Open wounds**

Database(s): Embase 1974 to 2020 May 20

1. exp Specimen Handling/ (1507)

2. (sampl* adj technique*).tw. (12136)

3. (sampl* adj approach*).tw. (2214)

4. (sampl* adj procedure*).tw. (4362)

5. (experiment* adj technique*).tw. (6149)

6. (experiment* adj approach*).tw. (21509)

7. (experiment* adj procedure*).tw. (8897)

8. 1 or 2 or 3 or 4 or 5 or 6 or 7 (56294)

9. exp Proteins/ (460479)

10. exp Enzymes/ (4027668)

11. exp Protease Inhibitors/ (375367)

12. exp Peptides/ (335857)

13. (protein* adj expres*).tw. (213064)

14. (protein* adj activit*).tw. (8330)

15. (protein* adj extract*).tw. (12135)

16. protein* inhibit* expres*.tw. (41)

17. protein* inhibit* activit*.tw. (280)

18. (proteas* or proteinas*).tw. (246069)

19. (proteas* adj (expres* or activit*)).tw. (16535)

20. ((up-regulation or down-regulation or accumulat* or reduct*) adj3 (proteas* or proteinas*)).tw. (1706)

21. 9 or 10 or 11 or 12 or 13 or 14 or 15 or 16 or 17 or 18 or 19 or 20 (4875962)

22. 8 or 21 (4922198)

23. exp Leg Ulcer/ (13176)

24. exp Foot Ulcer/ (5219)

25. exp decubitus/ (20558)

26. exp Diabetic Foot/ (15395)

27. exp penetrating trauma/ (12285)

28. exp Lacerations/ (11150)

29. exp Burns/ (67650)

30. exp Wound Infection/ (45035)

31. exp Abscess/ (99831)

32. exp Pilonidal Sinus/ (2350)

33. exp "Bites and Stings"/ (32647)

34. exp scar/ (73330)

35. exp Wound Healing/ (152413)

36. exp Soft Tissue Infections/ (11662)

37. ((plantar or diabetic or heel$ or foot or feet or ischaemic or ischemic or venous or varicose or stasis or arterial or decubitus or pressure or skin or leg or mixed) adj5 (wound$ or ulcer$)).tw. (68282)

38. (bedsore* or bed sore*).tw. (1016)

39. (pilonidal sinus* or pilonidal cyst*).tw. (1685)

40. (cavity wound* or sinus wound*).tw. (104)

41. (laceration* or gunshot or stab or stabbing or stabbed or bite*).tw. (68231)

42. (burn or burns or burned or scald*).tw. (74314)

43. (wound* adj5 dehisc*).tw. (7155)

44. (wound* adj5 infect*).tw. (47012)

45. (wound* adj5 disrupt*).tw. (1023)

46. wound complication*.tw. (7962)

47. (malignant wound* or experimental wound* or traumatic wound*).tw. (1425)

48. (infusion site* or donor site* or wound site*).tw. (21822)

49. (skin abscess* or skin abcess*).tw. (789)

50. exp exudate/ (9504)

51. (wound* adj3 (sampl* or fluid* or exudate* or discharge or effluent*)).tw. (4290)

52. or/23-50 (622895)

53. 51 and 52 (3263)

54. 22 and 53 (708)

55. human* volunteer*.tw. (10899)

56. volunt*.tw. (340620)

57. 55 or 56 (340620)

58. 54 and 57 (12)

59. (exp animal/ or animal.hw. or nonhuman/) not (exp human/ or human cell/ or (human or humans).ti.) (6384504)

60. 54 not 59 (521)

61. 58 or 60 (521)

**Ovid (Embase): Surgical wounds**

Database(s): Embase 1974 to 2020 May 18

1. exp sampling/ (436404)

2. (sampl* adj technique*).tw. (12130)

3. (sampl* adj approach*).tw. (2213)

4. (sampl* adj procedure*).tw. (4361)

5. (experiment* adj technique*).tw. (6144)

6. (experiment* adj approach*).tw. (21504)

7. (experiment* adj procedure*).tw. (8897)

8. 1 or 2 or 3 or 4 or 5 or 6 or 7 (485206)

9. exp protein/ (460467)

10. exp Enzymes/ (4026814)

11. exp Protease Inhibitors/ (375296)

12. exp Peptides/ (335821)

13. (protein* adj expres*).tw. (212991)

14. (protein* adj activit*).tw. (8327)

15. (protein* adj extract**).tw. (12134)

16. protein* inhibit* expres*.tw. (41)

17. protein* inhibit* activit*.tw. (280)

18. (proteas* or proteinas*).tw. (246014)

19. (proteas* adj (expres* or activit*)).tw. (16532)

20. ((up-regulation or down-regulation or accumulat* or reduct*) adj3 (proteas* or proteinas*)).tw. (1706)

21. 9 or 10 or 11 or 12 or 13 or 14 or 15 or 16 or 17 or 18 or 19 or 20 (4874995)

22. 8 or 21 (5282761)

23. exp Surgical Wound/ (6988)

24. exp surgical infection/ (45252)

25. exp Surgical Wound Dehiscence/ (17481)

26. exp exudate/ (9500)

27. exp wound drainage/ (12130)

28. (surg* adj5 infect*).tw. (47118)

29. (surg* adj5 wound*).tw. (20126)

30. (surg* adj5 site*).tw. (36321)

31. (surg* adj5 incision*).tw. (16244)

32. (surg* adj5 dehisc*).tw. (1688)

33. (wound* adj5 dehisc*).tw. (7152)

34. (wound* adj5 infect*).tw. (47000)

35. (wound adj5 disrupt*).tw. (883)

36. wound complication*.tw. (7962)

37. (drain* adj3 fluid*).tw. (7508)

38. or/23-37 (200157)

39. (wound* adj3 (sampl* or fluid* or exudate* or discharge or effluent*)).tw. (4290)

40. 22 and 38 and 39 (300)

41. (human* adj3 volunteer*).tw. (11994)

42. volunt*.tw. (340563)

43. 41 or 42 (340563)

44. 40 and 43 (8)

45. (exp animal/ or animal.hw. or nonhuman/) not (exp human/ or human cell/ or (human or humans).ti.) (6383543)

46. 40 not 45 (258)

47. 44 or 46 (258)

**EBSCO (CINAHL plus): Open wounds**

1. (MH "Specimen Handling+") (84363)

2. TI (sampl* W1 technique*) OR AB (sampl* W1 technique*) (2,972)

3. TI (sampl* W1 approach*) OR AB (sampl* W1 approach*) (607)

4. TI (sampl* W1 procedure*) OR AB (sampl* W1 procedure*) (872)

5. TI (experiment* W1 technique*) OR AB (experiment* W1 technique*) (219)

6. TI (experiment* W1 approach*) OR AB (experiment* W1 approach*) (1252)

7. TI (experiment* W1 procedure*) OR AB (experiment* W1 procedure*) (525)

8. 1 OR 2 OR 3 OR 4 OR 5 OR 6 OR 7 (90511)

9. (MH "Proteins+") (406276)

10. TI ( (proteas* or proteinas*) ) OR AB ( (proteas* or proteinas*) ) (8631)

11. TI ( (proteas* W1 (expres* or activit*)) ) OR AB ( (proteas* W1 (expres* or activit*)) ) (596)

12. (MH "Enzymes+") (124584)

13. TI ( ((up-regulation or down-regulation or accumulat* or reduct*) N3 (proteas* or proteinas*)) ) OR AB ( ((up-regulation or down-regulation or accumulat* or reduct*) N3 (proteas* or proteinas*)) ) (89)

14. (MH "Protease Inhibitors+") (19570)

15. (MH "Peptides+") (194568)

16. TI (protein* W1 expres*) OR AB (protein* W1 expres*) (13262)

17. TI (protein* W1 activit*) OR AB (protein* W1 activit*) (645)

18. TI (protein* W1 extract*) OR AB (protein* W1 extract*) (431)

19. TI "protein* inhibit* expres*" OR AB "protein* inhibit* expres*" (1)

20. TI "protein* inhibit* activit*" OR AB "protein* inhibit* activit*" (3)

21. 9 OR 10 OR 11 OR 12 OR 13 OR 14 OR 15 OR 16 OR 17 OR 18 OR 19 OR 20 (536027)

22. 8 OR 21 (612736)

23. (MH "Leg Ulcer+") (16222)

24. (MH "Foot Ulcer+") (10388)

25. (MH "Pressure Ulcer+") (15050)

26. (MH "Diabetic Foot") (9476)

27. (MH "Wounds, Penetrating+") (10856)

28. (MH "Tears and Lacerations+") (3949)

29. (MH "Burns+") (19179)

30. (MH "Wound Infection+") (15073)

31. (MH "Abscess+") (7740)

32. (MH "Pilonidal Cyst") (321)

33. (MH "Bites and stings+") (5363)

34. (MH "Cicatrix+") (6108)

35. (MH "Wound Healing+") (32521)

36. (MH "Soft Tissue Infections") (1446)

37. TI ( ((plantar or diabetic or heel* or foot or feet or ischaemic or ischemic or venous or varicose or stasis or arterial or decubitus or pressure or skin or leg or mixed) N5 (wound* or ulcer*)) ) OR AB ( ((plantar or diabetic or heel* or foot or feet or ischaemic or ischemic or venous or varicose or stasis or arterial or decubitus or pressure or skin or leg or mixed) N5 (wound* or ulcer*) ) (26957)

38. TI ( (bedsore* or bed sore*) ) OR AB ( (bedsore* or bed sore*) ) (329)

39. TI ( (pilonidal sinus* or pilonidal cyst*) ) OR AB ( (pilonidal sinus* or pilonidal cyst*) ) (272)

40. TI ( (cavity wound* or sinus wound*) ) OR AB ( (cavity wound* or sinus wound*) ) (275)

41. TI ( (laceration* or gunshot or stab or stabbing or stabbed or bite*) ) OR AB ( (laceration* or gunshot or stab or stabbing or stabbed or bite*) ) (13761)

42. TI ( (burn or burns or burned or scald*) ) OR AB ( (burn or burns or burned or scald*) ) (21352)

43. TI (wound* N5 dehisc*) OR AB (wound* N5 dehisc*) (1222)

44. TI (wound* N5 infect*) OR AB (wound* N5 infect*) (8575)

45. TI (wound* N5 disrupt*) OR AB (wound* N5 disrupt*) (180)

46. TI "wound complication*" OR AB "wound complication*" (1752)

47. TI ( (malignant wound* or experimental wound* or traumatic wound*) ) OR AB ( (malignant wound* or experimental wound* or traumatic wound*) ) (1046)

48. TI ( ("infusion site"* or "donor site"* or "wound site"*) ) OR AB ( ("infusion site"* or "donor site"* or "wound site"*) ) (2342)

49. TI (skin abscess* or skin abscess*) OR AB ( (skin abscess* or skin abscess*) (297)

50. (MH "Exudates and Transudates") (2708)

51. TI ( (wound* N3 (sampl* or fluid* or exudate* or discharge or effluent*)) ) OR AB ( (wound* N3 (sampl* or fluid* or exudate* or discharge or effluent*)) ) (1384)

52. 23 OR 24 OR 25 OR 26 OR 27 OR 28 OR 29 OR 30 OR 31 OR 32 OR 33 OR 34 OR 35 OR 36 OR 37 OR 38 OR 39 OR 40 OR 41 OR 42 OR 43 OR 44 OR 45 OR 46 OR 47 OR 48 OR 49 OR 50 (145358)

53. 51 AND 52 (1156)

54. 22 AND 53 (286)

55. TI (human* N3 volunteer*) OR AB (human* N3 volunteer*) (1068)

56. TI volunt* OR AB volunt* (68220)

57. 55 OR 56 (68220)

58. 54 AND 57 (5)

59. TI (animal model*) (3290)

60. MH animals+ (93485)

61. MH (animal studies) (137213)

62. 59 OR 60 OR 61 (222495)

63. (MH "Human") (2420826)

64. 62 NOT 63 (194450)

65. 54 NOT 64 (233)

66. 58 OR 65 (233)

67. 54 NOT 66 (53)

**EBSCO (CINAHL plus): Surgical wounds**

1. (MH "Specimen Handling+") (84609)

2. TI (sampl* W1 technique*) OR AB (sampl* W1 technique*) (2990)

3. TI (sampl* W1 approach*) OR AB (sampl* W1 approach*) (609)

4. TI (sampl* W1 procedure*) OR AB (sampl* W1 procedure*) (874)

5. TI (experiment* W1 technique*) OR AB (experiment* W1 technique*) (219)

6. TI (experiment* W1 approach*) OR AB (experiment* W1 approach*) (1254)

7. TI (experiment* W1 procedure*) OR AB (experiment* W1 procedure*) (526)

8. 1 OR 2 OR 3 OR 4 OR 5 OR 6 OR 7 (90780)

9. (MH "Proteins+") (407523)

10. (MH "Enzymes+") (124994)

11. (MH "Protease Inhibitors+") (19611)

12. (MH "Peptides+") (195101)

13. TI (protein* W1 expres*) OR AB (protein* W1 expres*) (13299)

14. TI (protein* W1 activit*) OR AB (protein* W1 activit*) (646)

15. TI (protein* W1 extract*) OR AB (protein* W1 extract*) (431)

16. TI "protein* inhibit* expres*" OR AB "protein* inhibit* expres*" (1)

17. TI "protein* inhibit* activit*" OR AB "protein* inhibit* activit*" (3)

18. TI ( (proteas* or proteinas*) ) OR AB ( (proteas* or proteinas*) ) (8649)

19. TI ( (proteas* W1 (expres* or activit*)) ) OR AB ( (proteas* W1 (expres* or activit*)) ) (597)

20. TI ( ((up-regulation or down-regulation or accumulat* or reduct*) N3 (proteas* or proteinas*)) ) OR AB ( ((up-regulation or down-regulation or accumulat* or reduct*) N3 (proteas* or proteinas*)) ) (89)

21. 9 OR 10 OR 11 OR 12 OR 13 OR 14 OR 15 OR 16 OR 17 OR 18 OR 19 OR 20 (537595)

22. 8 OR 21 (614531)

23. (MH "Surgical Wound") (1386)

24. (MH "Surgical Wound Infection") (11048)

25. (MH "Surgical Wound Dehiscence") (1581)

26. (MH "Exudates and Transudates") (2712)

27. (MH "Drainage+") (10418)

28. TI (surg* N5 infect*) OR AB (surg* N5 infect*) (11604)

29. TI (surg* N5 wound*) OR AB (surg* N5 wound*) (4694)

30. TI (surg* N5 site*) OR AB (surg* N5 site*) (9561)

31. TI (surg* N5 incision*) OR AB (surg* N5 incision*) (3014)

32. TI (surg* N5 dehisc*) OR AB (surg* N5 dehisc*) (420)

33. TI (wound* N5 dehisc*) OR AB (wound* N5 dehisc*) (1227)

34. TI (wound* N5 infect*) OR AB (wound* N5 infect*) (8584)

35. TI (wound N5 disrupt*) OR AB (wound N5 disrupt*) (178)

36. TI "wound complication" OR AB "wound complication" (361)

37. TI (drain* N3 fluid*) OR AB (drain* N3 fluid*) (968)

38. 23 OR 24 OR 25 OR 26 OR 27 OR 28 OR 29 OR 30 OR 31 OR 32 OR 33 OR 34 OR 35 OR 36 OR 37 (47510)

39. TI ( (wound* N3 (sampl* or fluid* or exudate* or discharge or effluent*)) ) OR AB ( (wound* N3 (sampl* or fluid* or exudate* or discharge or effluent*)) ) (1382)

40. 22 AND 38 AND S39 (139)

41. TI (human* N3 volunteer*) OR AB (human* N3 volunteer*) (1069)

42. TI volunt* OR AB volunt* (68333)

43. 41 OR 42 (68333)

44. 40 AND 43 (3)

45. TI (animal model*) (3295)

46. MH animals+ (93685)

47. MH (animal studies) (137708)

48. 45 OR 46 OR 47 (223178)

49. (MH "Human") (2427047)

50. 48 NOT 49 (194913)

51. 40 NOT 50 (129)

52. 44 OR 51 (129)

**Table S1.** Grouping of reported wound types into groups

| **Wound type (as reported)** | **Defined wound group** |
| --- | --- |
| Amputation wound | Amputation or traumatic wound |
| Bilateral lower-extremity amputation wounds |  |
| Combat-related extremity wound |  |
| Minor amputation |  |
| Traumatic bone fracture surgical wound |  |
| Traumatic ulcer |  |
| Traumatic wound |  |
| Arterial ulcer | Arterial ulcer |
| Artificially induced blister | Artificial wound |
| Artificial sunburn lesion |  |
| Artificial superficial skin wound |  |
| Full thickness skin biopsy |  |
| Punch biopsy |  |
| Burn blister | Burn wound |
| Burn wound |  |
| Frostbite blister |  |
| Full thickness burn |  |
| Partial thickness burn |  |
| Second degree burn |  |
| Bilateral mucogingival defect | Dental wound |
| Gingivectomy wound |  |
| Horizontal periodontal defect |  |
| Intrabony periodontal defect |  |
| Periodontal pocket |  |
| Periodontal surgical wound |  |
| Foot ulcer | Foot ulcer |
| Malum perforans wound |  |
| Mixed vessel disease ulcer | Mixed vessel disease ulcer |
| Acute cutaneous wound | Other wounds |
| Blister |  |
| Chronic fistula |  |
| Hidradenitis suppurativa |  |
| Injured soft tissue |  |
| Malignant fungating wound |  |
| Oncologic ulcer |  |
| Pilonidal abscess |  |
| Pyoderma gangrenosum wound |  |
| Soft tissue wound |  |
| Submandibular cyst |  |
| Systemic lupus erythematosus wound |  |
| Vasculitic ulcer |  |
| Decubitus ulcer | Pressure ulcer |
| Pressure ulcer |  |
| Abdominal surgery wound | Surgical wound  Surgical wound  Surgical wound |
| Abdominoplasty surgical wound |  |
| Ablation wound |  |
| Angioplasty surgical wound |  |
| Bariatric surgery wound |  |
| Brain aneurysm surgical wound |  |
| Branchial cyst surgical wound |  |
| Ceserean section |  |
| cholecystectomy surgical wound |  |
| Chronic surgical wound |  |
| Colectomy surgical wound |  |
| Colorectal cancer surgical wound |  |
| Coronary artery bypass graft |  |
| Cystectomy surgical wound |  |
| Disk protrusion surgical wound |  |
| Facial surgery wound |  |
| Flap surgery wound |  |
| Gastrectomy surgery wound |  |
| Gastrointestinal surgery wound |  |
| Hand reconstruction |  |
| Heminephrectomy surgical wound |  |
| Hernia wound |  |
| Hip replacement surgical wound |  |
| Hip surgery wound |  |
| Incisional hernia |  |
| Laminectomy surgical wound |  |
| Liposuction surgical wound |  |
| Lumpectomy surgical wound |  |
| Mammary benign disease surgical wound |  |
| Mammoplasty surgical wound |  |
| Mastectomy surgical wound |  |
| Mohs surgery wound |  |
| Molar removal wound |  |
| Musculoskeletal surgical wound |  |
| Neck dissection surgical wound |  |
| Nissen fundoplication surgical wound |  |
| Open head and neck surgical wound |  |
| Orthopedic surgical wound |  |
| Pancreatectomy surgical wound |  |
| Prostatectomy surgical wound |  |
| Proximal gastric vagotomy surgical wound |  |
| Quadrantectomy surgical wound |  |
| Radical neck dissection |  |
| Skin graft donor site |  |
| Spinal surgical wound |  |
| Surgical incision |  |
| Surgical wound |  |
| Sympathectomy surgical wound |  |
| Thyroid surgical wound |  |
| Total knee arthroplasty |  |
| TRAM Flap Breast Reconstruction |  |
| Tumor excision surgical wound |  |
| Ureterotomy surgical wound |  |
| Vagotomy surgical wound |  |
| Vestibuloplasty surgical wound |  |
| Unknown acute wound | Unknown wound |
| Unknown chronic wound |  |
| Venous leg ulcer | Venous leg ulcer |
| ^a^'Other wounds' were defined as those that could not be classed in any of the other wound groups | |

**Table S2.** Grouping of reported collection methods into groups

| **Collection method (as reported)** | **Defined collection method group** |
| --- | --- |
| Aspiration from beneath an occlusive dressing | Collection beneath occlusive dressing |
| Aspiration through occlusive dressing |  |
| Collection from beneath occlusive dressing |  |
| Withdrawal from underneath occlusive dressing using a micropipette |  |
| Aspiration of wound washout | Collection of wound washout |
| Collection of wound washout |  |
| Aspiration | Direct collection |
| Direct collection with glass microcapillary |  |
| Direct collection with micro-capillary tube |  |
| Direct collection with needle |  |
| Direct collection with spoon |  |
| Direct syringe collection |  |
| Direct collection using capillary pipette |  |
| Percutaneous aspiration |  |
| Centrifugation of PVA sponges applied to wound | Extraction from absorbent materials |
| Centrifugation of wound dressing |  |
| Elution from dressing in buffer |  |
| Elution from dressing in buffer under constant agitation |  |
| Elution from filter paper in buffer |  |
| Expressed from wound dressing in buffer |  |
| Expressed mechanically from wound dressing |  |
| Extraction from applied foam by washing |  |
| Extraction from collagen squares applied to wound |  |
| Extraction from cotton gauze |  |
| Extraction from dextranomer beads |  |
| Extraction from dressing |  |
| Extraction from filter paper |  |
| Extraction from nitrocellulose membrane |  |
| Extraction from paper strips |  |
| Extraction from polyester tipped applicators |  |
| Fluid squeezed out of dressing using syringe |  |
| Homogenisation of dressing in buffer |  |
| Lavaging dressing with saline |  |
| Rinsing out applied dressing |  |
| Squeezing dressing applied to wound |  |
| Squeezing sponges applied to wound |  |
| Withdrawal from dressing using micropipette |  |
| Withdrawal from dressing using syringe |  |
| Collection from debridement material | Other collection methods |
| Injection and withdrawal of sterile water from wound |  |
| Microdialysis |  |
| Suction collection |  |
| Levine swab technique | Swab technique |
| Serena swab technique |  |
| Swab technique |  |
| Unknown | Unknown collection method |
| 3 way stopcock device | Vacuum, drainage or other external device collection |
| Cellstick device |  |
| Closed-suction drainage device |  |
| Collection from sterile skin chambers |  |
| Collection in bag |  |
| Collection in sterile glove |  |
| Collection in sterile plastic sleeve |  |
| Negative pressure wound therapy cannister |  |
| Specialised low suction drain |  |
| Specimen trap integrated into negative pressure wound therapy device |  |
| Syringe collection from a surgical drainage tube |  |
| Syringe collection from cutaneous vinyl wound chambers |  |
| Syringe collection from implanted Silastic silicone rubber tubing |  |
| Syringe collection from plate attached to wound |  |
| Vacuum drainage device |  |
| Wound drainage device |  |
| ^a^'Other collection methods' were defined as those that could not be classed in any of the other collection method groups | |

**Table S3.** Grouping of reported assay methods into groups

| **Assay method (as reported)** | **Assay method group** |
| --- | --- |
| Absorbance assay | Absorbance assay |
| Bromcresol green method |  |
| Colometric assay |  |
| Electrochemiluminescent multiarray |  |
| Fluorometric assay |  |
| Spectrophotometry |  |
| Bioassay | Bioassay |
| Cytotoxicity assay |  |
| Cell migration assay | Cell-based assay |
| Flow cytometry |  |
| Fluorescence-activated cell sorting (FACS)^a^ |  |
| Turbidimeter |  |
| Gel filtration high performance liquid chromatography | Chromatography |
| Heparin affinity chromatography |  |
| HiTrap Protein A HP column |  |
| Ion exchange high performance liquid chromatography |  |
| Reversed phase high performance liquid chromatography |  |
| Trypsin Affinity Chromatography |  |
| Azocoll assay | Enzyme activity assay |
| Peptide substrate activity assay |  |
| 2-D DIGE | Gel electrophoresis |
| Electrophoresis and densitometry |  |
| Isoelectric focusing |  |
| LDS-PAGE |  |
| SDS-PAGE |  |
| Bead based multiplex immunoassay | Immunoassay  Immunoassay |
| Chemiluminescent immunoassay (CLIA) |  |
| Cytokine array |  |
| Cytometric bead array |  |
| Enzyme linked immunosorbent assay (ELISA) |  |
| Immunoassay |  |
| Immunocapture assay |  |
| Immunofluorescence |  |
| Immunostaining |  |
| Latex immunoagglutination assay |  |
| Multiplex ELISA array |  |
| Planar antibody array |  |
| Protein microarray |  |
| Radial immunodiffusion |  |
| Dot blot | Immunoblot |
| Western blot |  |
| iTRAQ Mass Spectrometry | Mass spectrometry |
| Liquid Chromatography Mass Spectrometry (LCMS) |  |
| Matrixassisted laser desorption/ionization–time of flight (MALDI-TOF) |  |
| SELDI-TOF Mass Spectrometry |  |
| Automated biochemical analyzer | Other assay methods |
| Edman degredation |  |
| Enzyme-dependent dye trapping system |  |
| Hemolysis assay |  |
| Nephelometry |  |
| X-gal–based staining |  |
| Filter aided sample preparation (FASP) | Protein processing method |
| Immunodepletion |  |
| Immunoprecipitation (IP) |  |
| Gamma scintillation counter | Radiation-based assay |
| Radioimmunoassay |  |
| Radioreceptor assay |  |
| Unknown | Unknown |
| Casein zymography | Zymography |
| Collagen zymography |  |
| Gelatin zymography |  |
| Zymography |  |
| ^a^'Other assay methods' were defined as those that could not be classed in any of the other method groups | |

**Table S4.** All relevant data extracted from included studies using the data charting form

| **Authors** | **Participant type** | **Setting** | **No. of  participants** | **Type of wound** | **Collection technique** | **Wound fluid  volume (ml)** | **Total protein  concentration (mg/ml)** | **Immediate  processing?** | **Sample  storage** | **No. of samples  collected (per wound)** | **Frequency of collection** | **No. of unique  proteins identified** | **Analytical techniques used** |
| --- | --- | --- | --- | --- | --- | --- | --- | --- | --- | --- | --- | --- | --- |
| Agarwal et al., 2013 | Patient (open wound), Patient (surgical wound) | Unknown | 20 | Venous leg ulcer, Surgical wound | Collection from beneath occlusive dressing | N/A | N/A | Yes | -80°c | 1 | N/A | 1 | Enzyme linked immunosorbent assay (ELISA) |
| Agren et al., 1998 | Patient (surgical wound) | Unknown | 15 | Hernia wound | Cellstick device | 1.3 | N/A | Yes | -70°c | 2 | Collected at 24 and 48 hours post-operatively | 2 | Enzyme linked immunosorbent assay (ELISA), Gelatin zymography |
| Ahmad et al., 2015 | Patient (open wound) | Wound healing center | 30 | Venous leg ulcer | Aspiration from beneath an occlusive dressing | N/A | 35.44 | Yes | -80°c | 1 | N/A | Fluorometric assay: 1, ELISA: 2 | Fluorometric assay, Enzyme linked immunosorbent assay (ELISA) |
| Aiba-Kojima et al., 2007 | Patient (surgical wound) | Hospital | 15 | Abdominoplasty surgical wound, Mammoplasty surgical wound, Liposuction surgical wound | Closed-suction drainage device | N/A | N/A | Yes | -80°c | Varied | On day 0 or 1 and day 5 or 6 after surgery | 13 | Enzyme linked immunosorbent assay (ELISA) |
| Akinci et al., 2014 | Patient (surgical wound) | Unknown | 54 | Colorectal cancer surgical wound | Closed-suction drainage device | N/A | 5.6 | Yes | -80°c | 3 | Collected on postoperative days 1–3 at 24-hour intervals. | 6 | Enzyme linked immunosorbent assay (ELISA) |
| Amato et al., 2015 | Patient (open wound) | Unknown | 45 | Venous leg ulcer | Aspiration through occlusive dressing | N/A | N/A | Yes | -20°c | 1 | N/A | 2 | Enzyme linked immunosorbent assay (ELISA) |
| Ambrosch et al., 2008 | Patient (open wound) | Hospital | 45 | Venous leg ulcer, Mixed vessel disease ulcer | Aspiration of wound washout | N/A | N/A |  |  |  |  | 2 | Chemiluminescent immunoassay (CLIA) |
| Ambrosch et al., 2014 | Patient (open wound) | Unknown | 0 | Diabetic foot ulcer | Extraction from filter paper | 0.59 | N/A | Yes | -20°c | 2 | All at once | 3 | Enzyme linked immunosorbent assay (ELISA) |
| Andres et al., 2003 | Patient (surgical wound) | Univeristy medical center | 20 | Total knee arthroplasty | Vacuum drainage device | N/A | N/A | Yes | -70°c | 5 | Collected immediately pre-operatively and at 1, 6, 24 and 48 hours after surgery | 3 | Enzyme linked immunosorbent assay (ELISA) |
| Arai et al., 2012 | Patient (surgical wound) | Univeristy medical center | 29 | Thyroid surgical wound | Wound drainage device | 16.38 | N/A | Yes | -80°c | 5 | Collected daily, 1-5 days post-operatively | Bead based multiplex immunoassay: 27, ELISA: 5 | Bead based multiplex immunoassay, Enzyme linked immunosorbent assay (ELISA) |
| Asadi et al., 2017 | Patient (open wound) | Hospital | 30 | Diabetic foot ulcer | Extraction from filter paper | 2 | N/A | Yes | -80°c | 4 | At the first and twelfth sessions after debridement before and immediately after the intervention. | 3 | Enzyme linked immunosorbent assay (ELISA) |
| Awad et al., 2012 | Patient (surgical wound) | Univeristy medical center | 14 | Coronary artery bypass graft | Wound drainage device | N/A | N/A | Yes | N/A | 3 | At 24, 48 and 72 hours post-surgery. | 4 | Enzyme linked immunosorbent assay (ELISA) |
| Baker and Leaper, 2000 | Patient (surgical wound) | Hospital | 50 | Mastectomy surgical wound, Colorectal cancer surgical wound | Closed-suction drainage device | N/A | N/A | Yes | -80°c | 1 | N/A | ELISA: 17, Gelatin zymography: 2 | Enzyme linked immunosorbent assay (ELISA), Gelatin zymography |
| Baker and Leaper, 2003 | Patient (surgical wound) | Hospital | 58 | Colorectal cancer surgical wound | Closed-suction drainage device | N/A | N/A | Yes | -80°c | Varied (up to 8) | Collected on the first post-operative day (18-24 hours after surgery) and every day following until the drain was removed. | Peptide substrate activity assay: 2, ELISA: 5 | Fluorometric assay, Enzyme linked immunosorbent assay (ELISA), Peptide substrate activity assay |
| Baker et al., 2003 | Patient (surgical wound) | Unknown | 52 | Colorectal cancer surgical wound | Closed-suction drainage device | N/A | N/A | Yes | -80°c | Varied: Up to 8 | Once a day for up to 8 days | 8 | Enzyme linked immunosorbent assay (ELISA) |
| Baker et al., 2006 | Patient (surgical wound) | Hospital | 44 | Colorectal cancer surgical wound | Closed-suction drainage device | N/A | N/A | Yes | -80°c | Varied | Collection started on post-operative day 1 until the drain was removed. | 3 | Enzyme linked immunosorbent assay (ELISA) |
| Baker et al., 2008 | Patient (surgical wound) | Hospital | 47 | Mastectomy surgical wound | Specialised low suction drain | 5 | N/A | Yes | -80°c | 3 | Collected at 24, 48 and 72 hours | Peptide substrate activity assay: 2, ELISA: 8 | Enzyme linked immunosorbent assay (ELISA), Peptide substrate activity assay |
| Banerjee et al., 2017 | Patient (open wound) | Unknown | 16 | Unknown chronic wound, Hidradenitis suppurativa | Levine swab technique | N/A | N/A | Yes | -80°c | 1 | N/A | 19 | Multiplex ELISA array |
| Barone et al., 1998 | Patient (open wound), Patient (surgical wound) | Unknown | 36 | Pressure ulcer, Abdominoplasty surgical wound, Abdominal surgery wound, Mammoplasty surgical wound, Flap surgery wound, Spinal surgical wound | Wound drainage device, Aspiration from beneath an occlusive dressing | N/A | N/A | No | 4°c | 1 | N/A | 3 | Enzyme linked immunosorbent assay (ELISA) |
| Bernatchez et al., 2013 | Patient (open wound), Patient (surgical wound) | Hospital, Medical center | 82 | Venous leg ulcer, Diabetic foot ulcer, Pressure ulcer, Surgical wound, Arterial ulcer, Burn wound | Swab technique | N/A | N/A | No | -79°c | 2 | Once before and once after cleansing. | 1 | Fluorometric assay, Immunocapture assay |
| Bodendorf et al., 2007 | Patient (surgical wound) | Unknown | 2 | Abdominal surgery wound | Wound drainage device | N/A | N/A | Yes | N/A | Varied | Varied | 1 | Reversed phase HPLC |
| Bodnar et al., 2018 | Patient (open wound) | Unknown | 30 | Venous leg ulcer, Diabetic foot ulcer, Second degree burn | Aspiration from beneath an occlusive dressing, Aspiration | N/A | N/A | Yes | -70°c | 1 | N/A | Spectrophotometry: 1, ELISA: 3 | Enzyme linked immunosorbent assay (ELISA), Spectrophotometry |
| Boeringer et al., 2018 | Patient (open wound) | Hospital | 11 | Unknown chronic wound | Extraction from filter paper | 0.15 | N/A | No | -80°c | 1 | N/A | 1 | Spectrophotometry, Peptide substrate activity assay |
| Bridges et al., 1987 | Patient (surgical wound) | Univeristy medical center | 11 | Mastectomy surgical wound | Closed-suction drainage device | N/A | N/A | Yes | -70°c | Varied | Samples were collected daily until the drain was removed. | 1 | Spectrophotometry, Rocket immunoelectrophoresis, Hemolysis assay |
| Broadbent et al., 2003 | Patient (surgical wound) | Hospital | 36 | Hernia wound | Closed-suction drainage device | N/A | N/A | Yes | -20°c | 1 | N/A | 3 | Enzyme linked immunosorbent assay (ELISA) |
| Broszczak et al., 2012 | Patient (open wound) | Community clinic | 64 | Venous leg ulcer, Diabetic foot ulcer, Pressure ulcer, Surgical wound, Mixed vessel disease ulcer | Aspiration from beneath an occlusive dressing, Levine swab technique | Foot ulcer: 0.5 | N/A | No | -80°c | Leg ulcers: Varied, Foot ulcers: 12 | Leg ulcers: Sequentially until ulcers healed or up to 24 weeks, Foot ulcers: Weekly | N/A | SELDI-TOF Mass Spectrometry, SDS-PAGE |
| Brown et al., 2011 | Patient (open wound) | Specialised medical center | 25 | Combat-related extremity wound | Negative pressure wound therapy cannister | 30 | N/A | No | -80°c | Varied: Until wound closure | Collected every 48 to 72 hours | 27 | Bead based multiplex immunoassay |
| Buchstein et al., 2009 | Patient (open wound) | Unknown | 22 | Venous leg ulcer, Acute cutaneous wound | Collection from beneath occlusive dressing | 1 | N/A | Yes | -80°c | 1 | N/A | 1 | Enzyme linked immunosorbent assay (ELISA), Spectrophotometry |
| Bullen et al., 1995 | Patient (surgical wound) | Unknown | 16 | Venous stasis ulcer, Mastectomy surgical wound | Wound drainage device, Rinsing out applied dressing | N/A | N/A | Yes | -70°c | Surgical wounds: Unknown, Open wounds: 1 | Surgical wounds: 1 hour after surgery and then every 12 hours until the drain was removed, Open wounds: N/A | Radioimmunoassay: 1, Gelatin zymography: 2 | Radioimmunoassay, Gelatin zymography |
| Cao et al., 2011 | Patient (open wound) | Hospital | 6 | Venous leg ulcer | Collection of wound washout | N/A | 0.855 | 0.855 | 0.855 | 0.855 | 0.855 | 4 | Fluorometric assay |
| Carvalho et al., 2010 | Patient (surgical wound) | Hospital | 38 | Ceserean section | 3 way stopcock device | 1.03 | N/A | Yes | -20°c | 5 | At 1,3,5,7 and 24 hours | 18 | Bead based multiplex immunoassay |
| Carvalho et al., 2010 | Healthy volunteer | Unknown | 11 | Artificial sunburn lesion | Microdialysis | N/A | N/A | No | -20°c | 2 | All at once | 36 | Bead based multiplex immunoassay |
| Carvalho et al., 2013 | Patient (surgical wound) | Hospital | 60 | Ceserean section | 3 way stopcock device | 1.03 | N/A | Yes | -20°c | 3 | Collected 4, 24 and 48 hours post-operatively | 9 | Bead based multiplex immunoassay |
| Caulfield et al., 2008 | Patient (open wound) | Burn center | 15 | Partial thickness burn | Extraction from collagen squares applied to wound | N/A | N/A | No | -80°c | 5 | Collected on days 1, 3, 5, 7 and 10. | 3 | Colometric assay |
| Cervero-Ferragut et al., 2017 | Patient (open wound), Patient (surgical wound) | Hospital | 47 | Pressure ulcer, Surgical wound | Negative pressure wound therapy cannister, Vacuum drainage device | N/A | N/A | No | -80°c | 5 | Collected on days 1, 3, 5, 7 and 10. | 3 | Turbidimeter |
| Chin et al., 2003 | Patient (open wound) | Unknown | 7 | Pressure ulcer | Aspiration from beneath an occlusive dressing | N/A | N/A | Yes | -80°c | 1 | N/A | 1 | Azocoll assay |
| Ching et al., 2005 | Patient (open wound), Patient (surgical wound) | Univeristy medical center | Unknown | Venous leg ulcer, Mohs surgery wound | Withdrawal from dressing using syringe | N/A | N/A | Yes | -70°c | 1 | N/A | 3 | Western blot |
| Chromy et al., 2013 | Patient (surgical wound) | Unknown | 19 | Combat-related extremity wound | Negative pressure wound therapy cannister | 30 | N/A | Yes | -80°c | Varied (44 total) | Collected every 48-72 hours until wound closure | 25 | Liquid Chromatography Mass Spectrometry (LCMS), 2-D DIGE |
| Chromy et al., 2014 | Patient (open wound) | Specialised medical center | 3 | Combat-related extremity wound | Negative pressure wound therapy cannister | 30 | N/A | Yes | -80°c | Unknown | Collected every 48-72 hours until surgical wound closure or coverage | 6 | SDS-PAGE, 2-D DIGE |
| Cooke et al., 2006 | Patient (surgical wound) | Dental clinic | 16 | Periodontal pocket | Extraction from paper strips | N/A | N/A | No | -80°c | 9 | Wound fluid was collected at baseline; between Days 3–5, 6–9, 12–15, 19–24; and at weeks 6, 12, 18, and 24 after reconstructive surgery. | ELISA: 2, Radioimmunoassay: 1 | Enzyme linked immunosorbent assay (ELISA), Radioimmunoassay |
| Cooper et al., 1994 | Patient (open wound) | Unknown | 20 | Pressure ulcer | Extraction from dextranomer beads | N/A | 39.45 | Yes | -80°c | 1 | N/A | 4 | Enzyme linked immunosorbent assay (ELISA) |
| Cowin et al., 2006 | Patient (open wound), Patient (surgical wound) | Wound healing center | 16 | Venous leg ulcer, Surgical wound | Wound drainage device, Unknown | N/A | N/A | Yes | -80°c | 1 | N/A | 1 | Enzyme linked immunosorbent assay (ELISA), Bioassay, Cytotoxicity assay |
| Cullen et al., 2002 | Patient (open wound) | Unknown | 9 | Diabetic foot ulcer | Elution from dressing in buffer | 1 | 6.67 | No | -70°c | 1 | N/A | Fluorometric assay: 3, Gelatin zymography: 2 | Fluorometric assay, Gelatin zymography |
| Cullen et al., 2002 | Patient (open wound) | Unknown | 3 | Diabetic foot ulcer | Elution from dressing in buffer | N/A | N/A | No | -70°c | 1 | N/A | Fluorometric assay: 1, Gelatin zymography: 2 | Fluorometric assay, Gelatin zymography |
| Dalhoff et al., 1982 | Patient (surgical wound) | Hospital | 28 | cholecystectomy surgical wound, Abdominal surgery wound, Vagotomy surgical wound, Angioplasty surgical wound, Sympathectomy surgical wound, Pancreatectomy surgical wound, Prostatectomy surgical wound, Ureterotomy surgical wound | Wound drainage device | N/A | 38 | Yes | N/A | 1 | N/A | 4 | Electrophoresis and densitometry |
| De Mattei et al., 2008 | Patient (open wound) | Unknown | 3 | Venous leg ulcer | Withdrawal from dressing using syringe | N/A | N/A | Yes | -80°c | 1 | N/A | 1 | X-gal–based staining, Immunofluorescence |
| Debats et al., 2006 | Patient (surgical wound) | Hospital | 30 | Skin graft donor site, Chronic surgical wound | Negative pressure wound therapy cannister, Centrifugation of PVA sponges applied to wound | 0.5 | N/A | Yes | -80°c | 1 | N/A | 1 | Enzyme linked immunosorbent assay (ELISA) |
| Debats et al., 2009 | Patient (surgical wound) | Hospital | 10 | Skin graft donor site | Centrifugation of PVA sponges applied to wound | 0.5 | N/A | Yes | -80°c | 3 (where possible) | Collection on days 2, 5 and 10 post-operatively | 1 | Enzyme linked immunosorbent assay (ELISA), Western blot |
| Deitch and Emmett, 1986 | Patient (open wound) | Burn center | 20 | Burn blister | Aspiration | N/A | N/A | If possible | -70°c | 1 | N/A | 19 | Crossed immunoelectrophoresis |
| Deitch et al., 1990 | Patient (open wound) | Univeristy medical center | 16 | Burn blister | Aspiration | N/A | N/A | If possible | -70°c | 1 | N/A | 2 | Flow cytometry |
| Dellavia et al., 2019 | Patient (open wound) | Unknown | 36 | Intrabony periodontal defect, Horizontal periodontal defect | Extraction from paper strips | 0.1 | N/A | No | -20°c | 5 | At baseline 4, 7 , 14 and 21 days after surgery | 1 | Enzyme linked immunosorbent assay (ELISA) |
| Di Vita et al., 2006 | Patient (surgical wound) | Unknown | 10 | Incisional hernia | Closed-suction drainage device | N/A | 42.7 | Yes | -70°c | 4 | Once a day | 7 | Enzyme linked immunosorbent assay (ELISA) |
| Di Vita et al., 2006 | Patient (surgical wound) | Unknown | 20 | Incisional hernia | Wound drainage device | N/A | N/A | Yes | -70°c | 4 | Collected on days 1-4 post-operatively | 6 | Enzyme linked immunosorbent assay (ELISA) |
| Drinkwater et al., 2003 | Patient (open wound), Patient (surgical wound) | Hospital | 40 | Venous leg ulcer, Mastectomy surgical wound | Wound drainage device, Aspiration from beneath an occlusive dressing | N/A | N/A | Yes | -80°c | 1 | N/A | 2 | Enzyme linked immunosorbent assay (ELISA) |
| Driver et al., 2013 | Patient (open wound) | Univeristy medical center | 17 | Diabetic foot ulcer | Extraction from filter paper | 0.1 | N/A | No | -80°c | 4 | Once a week | 15 | Bead based multiplex immunoassay |
| Edsberg et al., 2012 | Patient (open wound) | Unknown | 32 | Pressure ulcer | Extraction from polyester tipped applicators | 0.5 | 16.71 | Yes | -80°c | 15 (if possible) | On days 0, 1, 2, 3, 4, 7, 8, 9, 10, 11, 14, 21, 28, 35, and 42 (If possible) | Bead based multiplex immunoassay: 10, Planar antibody array: 40, iTRAQ Mass Spectrometry: 381 | Bead based multiplex immunoassay, iTRAQ Mass Spectrometry, Planar antibody array |
| Edsberg et al., 2015 | Patient (open wound) | Nursing facility | 36 | Pressure ulcer | Extraction from polyester tipped applicators | 0.255 | 5.6 | Yes | -20°c for under a week and then -80°c | Varies based on wound size | N/A | 22 | Bead based multiplex immunoassay |
| Edwards et al., 1999 | Patient (open wound) | Unknown | 5 | Pressure ulcer | Aspiration from beneath an occlusive dressing | N/A | N/A | Yes | N/A | 1 | N/A | 1 | Spectrophotometry |
| Edwards et al., 2001 | Patient (open wound) | Unknown | 1 | Pressure ulcer | Negative pressure wound therapy cannister | 3 | N/A | Yes | N/A | 1 | N/A | 1 | Spectrophotometry, Peptide substrate activity assay |
| Eming et al., 2008 | Patient (open wound) | Clinic | 6 | Venous leg ulcer | Collection from beneath occlusive dressing | 0.35 | N/A | Yes | -80°c | 1 | N/A | 2 | Gelatin zymography, Peptide substrate activity assay |
| Eming et al., 2010 | Patient (open wound), Patient (surgical wound) | Unknown | 28 | Venous leg ulcer, Surgical incision | Collection from beneath occlusive dressing | 1 | N/A | Yes | -80°c | 1 | N/A | ELISA: 2, Radioimmunoassay: 1, LCMS: 125, Dot blot: 4 | Enzyme linked immunosorbent assay (ELISA), Liquid Chromatography Mass Spectrometry (LCMS), Dot blot |
| Evans et al., 2012 | Patient (open wound) | Specialised medical center | 24 | Combat-related extremity wound | Negative pressure wound therapy cannister | 30 | N/A | No | -80°c | Varied: Until wound closure | Collected every 48 to 72 hours | 22 | Bead based multiplex immunoassay |
| Evans et al., 2014 | Patient (open wound) | Specialised medical center | 34 | Combat-related extremity wound | Negative pressure wound therapy cannister | N/A | N/A | No | -80°c | 2 | Collected once after initial debridement and once after the final debridement before wound closure. | 1 | Enzyme linked immunosorbent assay (ELISA) |
| Fazekas et al., 2019 | Patient (surgical wound) | Unknown | 5 | Vestibuloplasty surgical wound | Extraction from paper strips | N/A | N/A | No | -80°c | 14 | Each day for 14 post-operative days | 1 | Enzyme linked immunosorbent assay (ELISA) |
| Fernandez et al., 2008 | Patient (open wound) | Hospital, Nursing facility | 5 | Venous leg ulcer | Collection of wound washout | 1 | N/A | Yes | -80°c | 1 | N/A | LCMS: 16, MALDI-TOF: 24, Western blot: 3 | Liquid Chromatography Mass Spectrometry (LCMS), Matrix‐assisted laser desorption/ionization–time of flight (MALDI-TOF), Western blot |
| Fernandez et al., 2011 | Patient (open wound) | Hospital, Nursing facility | 29 | Venous leg ulcer | Collection of wound washout | 1 | N/A | Yes | -80°c | Varied | Wound fluid samples were collected from patients at various time points during the course of treatment depending on patient attendance, wound infection and availability of sample. | 1 | Western blot, Reversed phase HPLC |
| Fivenson et al., 1997 | Patient (open wound) | Unknown | 10 | Venous stasis ulcer | Homogenisation of dressing in buffer | 2 | N/A | Yes | N/A | 9 | Once a week | 16 | Enzyme linked immunosorbent assay (ELISA) |
| Flohé et al., 2007 | Patient (surgical wound) | Hospital | 21 | Hip surgery wouund | Wound drainage device | N/A | 66.5 | Yes | -80°c | 2 | Collected at 3 and 6 hours after surgery. | ELISA: 3, Western blot/FACS: 1, Cytometric bead array: 2 | Enzyme linked immunosorbent assay (ELISA), Western blot, Cytometric bead array, Fluorescence-activated cell sorting (FACS) |
| Forsberg et al., 2008 | Patient (open wound) | Other | 20 | Combat-related extremity wound | Direct syringe collection | 3 | N/A | Yes | -16°c | 1 | N/A | Bead based multiplex immunoassay: 22, ELISA: 1 | Bead based multiplex immunoassay, Enzyme linked immunosorbent assay (ELISA) |
| Frohm et al., 1996 | Patient (open wound), Patient (surgical wound) | Hospital | 8 | Venous leg ulcer, Blister, Abdominal surgery wound, Pyoderma gangrenosum wound, Systemic lupus erythematosus wound | Aspiration, Direct collection with spoon, Direct collection with micro-capillary tube | Abdominal surgery wound: 3.5, Blister: 4, Pyoderma gangrenosum wound: 1, Systemic lupus erythematosus wound: 0.3, Venous leg ulcer: 4 | N/A | No | -70°c | 1 | N/A | Western blot: 1, Edman degredation: 7 | Western blot, Edman degredation |
| Gallo et al., 1997 | Patient (surgical wound) | Hospital | 0 | Mastectomy surgical wound | Closed-suction drainage device | N/A | N/A | Yes | -20°c | 1 | N/A | 1 | Spectrophotometry, Absorbance assay |
| Ganesh et al., 2012 | Patient (open wound), Patient (surgical wound) | Wound healing center | 15 | Pressure ulcer, Surgical wound | Lavaging dressing with saline | N/A | N/A | Yes | N/A | 1 | N/A | 1 | Enzyme linked immunosorbent assay (ELISA) |
| Gohel et al., 2008 | Patient (open wound) | Wound healing center | 80 | Venous leg ulcer | Aspiration from beneath an occlusive dressing | 0.23 | N/A | Yes | -80°c | 2 (if possible) | Collected at week 0 and at week 4-6 (if possible). | ELISA: 5, Gelatin zymography: 2 | Enzyme linked immunosorbent assay (ELISA), Gelatin zymography |
| Goto et al., 2016 | Patient (open wound) | Hospital | 13 | Venous leg ulcer | Elution from dressing in buffer | 10 | N/A | Yes | -80°c | Varied | Varied | 2 | Enzyme linked immunosorbent assay (ELISA) |
| Gottrup et al., 2013 | Patient (open wound) | Wound healing center | 39 | Diabetic foot ulcer | Elution from dressing in buffer | N/A | N/A | No | -70°c | 2 | Collected at week 0 and week 4 | 4 | Fluorometric assay, Protein microarray |
| Grande et al., 2020 | Patient (surgical wound) | Unknown | 26 | Minor amputation | Aspiration through occlusive dressing | N/A | N/A | Yes | -70°c | 4 | Collected 3 days after vein graft and thereafter at 1, 3, and 5 months. | 6 | Enzyme linked immunosorbent assay (ELISA) |
| Grayson et al., 1993 | Patient (surgical wound) | Burn center | 13 | Skin graft donor site | Aspiration from beneath an occlusive dressing | N/A | N/A | Yes | -70°c | Varied | Collected on post-operative day 1 onwards every 24 hours until no further fluid could be obtained. | 5 | Enzyme linked immunosorbent assay (ELISA) |
| Grieb et al., 2012 | Patient (open wound), Patient (surgical wound) | Hospital | 33 | Diabetic foot ulcer, Pressure ulcer, Surgical wound | Wound drainage device, Elution from dressing in buffer | N/A | N/A | Yes | -80°c | 1 | N/A | 1 | Enzyme linked immunosorbent assay (ELISA) |
| Grimstad et al., 2011 | Patient (surgical wound) | Unknown | 5 | Mammoplasty surgical wound | Wound drainage device | N/A | N/A | No | Unknown | 1 | N/A | 25 | Bead based multiplex immunoassay |
| Grinnell and Zhu, 1994 | Patient (open wound) | Hospital | 11 | Partial thickness burn, Full thickness burn | Collection in sterile glove | 7.5 | N/A | Yes | -70°c | 1 | N/A | 1 | Western blot, Spectrophotometry, Gelatin zymography |
| Grinnell and Zhu, 1996 | Patient (open wound), Patient (surgical wound) | Unknown | 4 | Venous stasis ulcer, Mastectomy surgical wound | Aspiration from beneath an occlusive dressing, Wound drainage device | N/A | N/A | Yes | -70°c | 4 | Once a week for one month. | Western blot: 3, Spectrophotometry: 1, Gelatin zymography: 2 | Western blot, Spectrophotometry, Gelatin zymography |
| Grinnell et al., 1992 | Healthy volunteer, Patient (open wound), Patient (surgical wound) | Hospital | 19 | Venous leg ulcer, Artificially induced blister, Mastectomy surgical wound | Closed-suction drainage device, Collection from beneath occlusive dressing | N/A | N/A | Yes | -70°c | 1 | N/A | 2 | Western blot |
| Grinnell et al., 1993 | Patient (open wound) | Hospital | 11 | Partial thickness burn, Full thickness burn | Collection in sterile glove | 7.5 | 36 | Yes | -70°c | 1 | N/A | Western blot: 3, Gelatin zymography: 1 | Western blot, Gelatin zymography |
| Grinnell et al., 1998 | Patient (open wound), Patient (surgical wound) | Unknown | 11 | Venous stasis ulcer, Mastectomy surgical wound, Burn wound | Closed-suction drainage device, Aspiration from beneath an occlusive dressing, Collection in sterile glove | N/A | Burn wound: 37.5 | Yes | -70°c | Surgical wounds: 2, Blister wounds: Up to 7, Open wounds: 3 | Surgical wounds: 24 and 48 hours after surgery. Blister wounds: Once 4-8 hours after injury and then every 48-72 hrs. Open wounds: Weekly | 3 | Western blot, SDS-PAGE |
| Gronberg et al., 2011 | Patient (open wound) | Wound healing center | 8 | Venous leg ulcer | Collection from beneath occlusive dressing | N/A | 42.5 | Yes | -80°c | 1 | N/A | Spectrophotometry/Gelatin zymography: 1, Western blot: 2 | Spectrophotometry, Western blot, Gelatin zymography |
| Grzela et al., 2014 | Patient (open wound) | Unknown | 8 | Venous leg ulcer | Aspiration from beneath an occlusive dressing | 0.5 | N/A | Yes | N/A | 3 | Collected at the beginning of the study (day 0) and after 14 and 28 days of treatment | 2 | Gelatin zymography |
| Hardwicke et al., 2010 | Patient (open wound), Patient (surgical wound) | Hospital | 10 | Venous leg ulcer, Mastectomy surgical wound | Closed-suction drainage device, Elution from dressing in buffer under constant agitation | Mastectomy surgical wound: 1 | Mastectomy surgical wound: 4.756, Venous leg ulcer: 1.276 | Yes | -80°c | 1 | N/A | 1 | Fluorometric assay, Enzyme linked immunosorbent assay (ELISA), Spectrophotometry |
| Harris et al., 1995 | Patient (open wound) | Unknown | 18 | Venous leg ulcer | Aspiration from beneath an occlusive dressing | N/A | 13.0125 | Yes | -70°c | 1 | N/A | Bioassay/Nephelometry: 1, ELISA: 4 | Nephelometry, Enzyme linked immunosorbent assay (ELISA), Bioassay |
| Hasmann et al., 2011 | Patient (open wound), Patient (surgical wound) | Unknown | 26 | Surgical wound, Decubitus ulcer, Blister | Negative pressure wound therapy cannister, Direct collection with needle, Direct collection with spoon | N/A | N/A | Yes | -20°c | 1 | N/A | Fluorometric assay/Spectrophotometry/Turbidimeter/Western blot: 1, Gelatin zymography: 2 | Fluorometric assay, Western blot, Spectrophotometry, Turbidimeter, Zymography |
| Hasmann et al., 2011 | Patient (open wound), Patient (surgical wound) | Unknown | 27 | Venous leg ulcer, Pressure ulcer, Surgical wound, Blister, Malum perforans wound | Negative pressure wound therapy cannister, Direct collection with needle, Direct collection with spoon | N/A | N/A | Yes | N/A | 1 | N/A | 2 | Peptide substrate activity assay |
| Hasmann et al., 2013 | Patient (open wound), Patient (surgical wound) | Unknown | 27 | Venous leg ulcer, Diabetic foot ulcer, Decubitus ulcer, Blister, Surgical wound | Negative pressure wound therapy cannister, Direct collection with needle, Direct collection with spoon | N/A | N/A | Yes | -20°c | 1 | N/A | 2 | Spectrophotometry |
| Haukipuro et al., 1987 | Patient (surgical wound) | Hospital | 18 | Proximal gastric vagotomy surgical wound, Nissen fundoplication surgical wound, cholecystectomy surgical wound | Syringe collection from implanted Silastic silicone rubber tubing | 0.183 | N/A | No | -20°c | 5 (if possible) | Once a day for 5 days (if possible) | 1 | Radioimmunoassay |
| Haukipuro et al., 1991 | Patient (surgical wound) | Hospital | 20 | Colorectal cancer surgical wound | Syringe collection from implanted Silastic silicone rubber tubing | 1 | N/A | No | -20°c | 7 | Collected daily for 7 days | Radioimmunoassay: 2, GF-HPLC: 1 | Radioimmunoassay, Gel filtration high performance liquid chromatography |
| Haukipuro, 1991 | Patient (surgical wound) | Hospital | 32 | Colorectal cancer surgical wound, Gastrointestinal surgery wound | Syringe collection from implanted Silastic silicone rubber tubing | 1 | N/A | No | -20°c | 7 (if possible) | Collected daily for 7 days or until discharge | 2 | Radioimmunoassay |
| Hawksworth et al., 2009 | Patient (open wound) | Unknown | 33 | Combat-related extremity wound | Negative pressure wound therapy cannister | 30 | N/A | No | -80°c | Varied: Until wound closure | Collected every 48 to 72 hours | 22 | Bead based multiplex immunoassay |
| He et al., 1998 | Patient (open wound) | Unknown | 4 | Burn wound | Collection in sterile glove | N/A | 50 | Yes | -70°c | Unknown | Sampling was initiated 4–8 h after injury (day 0) and continued at 48–72 h intervals. | 1 | Western blot, Spectrophotometry, Casein zymography, Gel filtration high performance liquid chromatography, Ion exchange high performance liquid chromatography, Immunoprecipitation (IP) |
| Heinzle et al., 2013 | Patient (open wound) | Unknown | 29 | Venous leg ulcer, Pressure ulcer, Surgical wound, Malum perforans wound | Swab technique | N/A | N/A | Unknown | N/A | 1 | N/A | Fluorometric assay: 2, Gelatin zymography: 1 | Fluorometric assay, Spectrophotometry, Gelatin zymography |
| Henshaw et al., 2014 | Patient (open wound) | Hospital | 24 | Diabetic foot ulcer | Extraction from paper strips | 0.125 | N/A | No | -80°c | Varied | Collected from week 0 to week 6 or until the ulcer healed (average time between visits was 10.5 days) | 2 | Gelatin zymography |
| Henshaw et al., 2015 | Patient (open wound) | Wound healing center | 32 | Diabetic foot ulcer | Extraction from filter paper | N/A | N/A | No | 4°c | 1 | N/A | 1 | Western blot |
| Hodde et al., 2020 | Patient (open wound) | Unknown | 10 | Venous leg ulcer | Elution from dressing in buffer | N/A | N/A | Yes | -80°c | Unknown | Once a week | ELISA: 1, Multiplex ELISA array: 16 | Enzyme linked immunosorbent assay (ELISA), Multiplex ELISA array |
| Hoffman et al., 1998 | Patient (open wound), Patient (surgical wound) | Unknown | 6 | Venous leg ulcer, Mastectomy surgical wound | Wound drainage device, Squeezing dressing applied to wound | Mastectomy surgical wound: 53.5, Venous leg ulcer: 11.8 | N/A | Yes | -70°c | 1 | N/A | Western blot: 2 , Spectrophotometry/Peptide substrate activity assay: 1 | Western blot, Spectrophotometry, SDS-PAGE, Peptide substrate activity assay |
| Hoffman et al., 1999 | Patient (open wound) | Wound healing center | 7 | Venous leg ulcer | Expressed from wound dressing in buffer | N/A | N/A | Yes | -70°c | 1 | N/A | 1 | Spectrophotometry, Peptide substrate activity assay |
| Holzheimer and Steinmetz, 2000 | Patient (surgical wound) | Unknown | 28 | Mammoplasty surgical wound | Closed-suction drainage device | N/A | N/A | Yes | -80°c | 36 | Collected pre-operatively, on the day of operation and post-operatively until the 7th post-operative day. Samples were collected 4 times a day (10 am, 2 pm, 6 pm, 10 pm). | 4 | Enzyme linked immunosorbent assay (ELISA) |
| Hormbrey et al., 2003 | Patient (surgical wound) | Hospital | 52 | Mastectomy surgical wound, Mammoplasty surgical wound | Wound drainage device | N/A | N/A | Unknown | N/A | 13 | Collected from post-surgical day 1 onwards until day 13 | 1 | Enzyme linked immunosorbent assay (ELISA) |
| Hourigan et al., 2010 | Patient (open wound), Patient (surgical wound) | Specialised medical center | 17 | Abdominal surgery wound, Soft tissue wound | Specimen trap integrated into negative pressure wound therapy device | 20 | Abdominal surgery wound: 2.9, Soft tissue wound: 2.59 | Yes | -70°c | 6 if possible | Collected on days 1, 2, 3 and then every other day up until day 9 or until NPWT was discontinued. | 4 | Automated biochemical analyzer |
| Huttunen and Harvima, 2005 | Patient (open wound) | Hospital | 16 | Venous leg ulcer, Venous stasis ulcer | Collection of wound washout | 1 | N/A | Yes | Unknown | 1 | N/A | 4 | Peptide substrate activity assay |
| Ihlberg et al., 1993 | Patient (surgical wound) | Hospital | 10 | Artificially induced blister, Surgical wound | Direct syringe collection, Syringe collection from implanted Silastic silicone rubber tubing | Artificially induced blister: 0.8, Surgical wound: 10 | N/A | Unknown | N/A | Blister wound: 5, Surgical wound: 7 | Blister wound: 1 day before operation and 1, 2, 4 and 7 days after operation. Surgical wound: Once a day post-op | 2 | Radioimmunoassay |
| Iizaka et al., 2010 | Patient (open wound) | Hospital, Univeristy medical center, Nursing facility | 28 | Pressure ulcer | Withdrawal from underneath occlusive dressing using a micropipette | 0.47 | 49 | Yes | -80°c | 1 | N/A | 1 | Colometric assay |
| Ingram et al., 2018 | Patient (open wound) | Community clinic | 61 | Diabetic foot ulcer | Swab technique | 3 | N/A | Yes | -80°c | 2 | Collected once at baseline and after 1 week. | 1 | Enzyme linked immunosorbent assay (ELISA) |
| Inoue et al., 1996 | Patient (open wound) | Hospital | 8 | Burn blister | Aspiration | N/A | N/A | Yes | -80°c | 1 | N/A | Nephelometry: 2, ELISA: 9, Bromcresol green method: 1, Latex immunoagglutination assay: 3 | Nephelometry, Enzyme linked immunosorbent assay (ELISA), Bromcresol green method, Latex immunoagglutination assay |
| Jafari et al., 2017 | Patient (open wound) | Hospital | 8 | Traumatic wound, Burn wound | Negative pressure wound therapy cannister, Collection from beneath occlusive dressing | N/A | N/A | No | -80°c | 1 | N/A | 10 | Bead based multiplex immunoassay |
| James et al., 2000 | Patient (open wound) | Community clinic | 12 | Venous leg ulcer, Mixed vessel disease ulcer | Aspiration from beneath an occlusive dressing | 0.5 | 37 | Yes | -70°c | 1 or 2 | Collected one at day 0 and once 4-10 weeks later (median 8 weeks) | 2 | Automated biochemical analyzer |
| James et al., 2003 | Patient (open wound), Patient (surgical wound) | Community clinic | 18 | Venous leg ulcer, Surgical wound, Mixed vessel disease ulcer, Mastectomy surgical wound, Incisional hernia, Hand reconstruction | Wound drainage device, Aspiration from beneath an occlusive dressing | N/A | N/A | Yes | -70°c | 1 | N/A | 1 | Enzyme linked immunosorbent assay (ELISA) |
| Jeganathan et al., 2012 | Patient (open wound) | Wound healing center | 10 | Venous leg ulcer | Elution from dressing in buffer under constant agitation, Swab technique | 12.5 | 0.7 | Yes | -80°c | 1 | N/A | 1 | Fluorometric assay |
| Jindatanmanusan et al., 2018 | Patient (open wound) | Hospital | 22 | Diabetic foot ulcer | Extraction from paper strips | 1 | N/A | Yes | -20°c | 5 | Collected at week 0, 1, 4, 8 and 12 | 1 | Enzyme linked immunosorbent assay (ELISA) |
| Kainulainen et al., 1998 | Patient (surgical wound) | Hospital | 0 | Mammoplasty surgical wound | Closed-suction drainage device | N/A | N/A | Yes | -70°c | 3 | Collected at 1 day intervals for 3 days | 2 | Fluorometric assay, Dot blot, Peptide substrate activity assay, Immunoprecipitation (IP) |
| Kaner et al., 2017 | Patient (surgical wound) | Dental clinic | 30 | Periodontal surgical wound | Extraction from paper strips | N/A | N/A | No | -80°c | 5 | Before surgery and 1, 3, 7 and 14 days after surgery | 4 | Bead based multiplex immunoassay |
| Karayiannakis et al., 2003 | Patient (surgical wound) | Hospital | 37 | Abdominal surgery wound, Incisional hernia | Closed-suction drainage device | N/A | N/A | Yes | -70°c | 5 | Collected 8 h after the operation (baseline) and thereafter on days 1, 3, 5 and 7 after operation | 1 | Enzyme linked immunosorbent assay (ELISA) |
| Keskiner et al., 2016 | Patient (surgical wound) | Dental clinic | 30 | Periodontal surgical wound | Extraction from paper strips | 0.3 | N/A | No | -80°c | 4 | 2 strips used on both day 7 and 12 post-surgery | 3 | Enzyme linked immunosorbent assay (ELISA) |
| Kilpadi et al., 2006 | Patient (open wound) | Unknown | 8 | Pressure ulcer | Collection from beneath occlusive dressing | N/A | N/A | Yes | -80°c | 4 | At day 0, 1, 3 and 7 of treatment | 5 | Enzyme linked immunosorbent assay (ELISA) |
| Kitamura et al., 2019 | Patient (open wound) | Hospital | 21 | Pressure ulcer | Extraction from nitrocellulose membrane | N/A | N/A | Unknown | N/A | 4 | On post-wounding days 1, 4, 7 and 10 | 1 | Unknown |
| Kloeters et al., 2015 | Patient (open wound) | Unknown | 33 | Pressure ulcer | Extraction from dressing | N/A | N/A | Unknown | N/A | 4 | On post-wounding days 1, 4, 7 and 10 | 1 | Fluorometric assay, Spectrophotometry, Peptide substrate activity assay |
| Koivukangas et al., 2005 | Healthy volunteer, Patient (other) | Unknown | 41 | Artificially induced blister | Aspiration | N/A | N/A | Unknown | N/A | 1 | Once per blister | 2 | Radioimmunoassay |
| Koschwanez et al., 2017 | Patient (surgical wound) | Hospital | 51 | Bariatric surgery wound | Wound drainage device | N/A | N/A | Yes | -80°c | 1 | N/A | 3 | Bead based multiplex immunoassay |
| Krishnaswami et al., 2002 | Patient (surgical wound) | Burn center | 10 | Skin graft donor site | Aspiration from beneath an occlusive dressing | N/A | N/A | Yes | -70°c | 1 | N/A | ELISA: 3, Spectrophotometry: 1 | Enzyme linked immunosorbent assay (ELISA), Spectrophotometry |
| Krisp et al., 2011 | Patient (open wound) | Hospital | 3 | Diabetic foot ulcer | Centrifugation of PVA sponges applied to wound | 2 | N/A | Yes | -80°c | 1 | N/A | 104 | Liquid Chromatography Mass Spectrometry (LCMS) |
| Krisp et al., 2012 | Patient (open wound) | Hospital | 0 | Diabetic foot ulcer, Burn wound | Extraction from applied foam by washing | N/A | N/A | Yes | -80°c | 1 | N/A | 25 | Liquid Chromatography Mass Spectrometry (LCMS) |
| Krisp et al., 2013 | Patient (open wound), Patient (surgical wound) | Hospital | 16 | Diabetic foot ulcer, Skin graft donor site | Centrifugation of PVA sponges applied to wound | 2 | N/A | Yes | -80°c | 1 | N/A | Western blot/Gelatin zymography: 2, LCMS: 714 | Western blot, Gelatin zymography, Liquid Chromatography Mass Spectrometry (LCMS), SDS-PAGE |
| Kulcenty et al., 2020 | Patient (surgical wound) | Specialised medical center | 38 | Lumpectomy surgical wound | Wound drainage device | N/A | N/A | Yes | -80°c | 1 | N/A | 50 | Multiplex ELISA array |
| Labler et al., 2009 | Patient (open wound) | Hospital | 32 | Soft tissue wound | Squeezing dressing applied to wound | N/A | N/A | Yes | -80°c | Varied | Varied | 4 | Enzyme linked immunosorbent assay (ELISA) |
| Ladwig et al., 2002 | Patient (open wound) | Unknown | 56 | Pressure ulcer | Extraction from dextranomer beads | 1 | N/A | Yes | -80°c | 3 | Collected on day 0 (pre-treatment), day 10 and day 36. | ELISA: 1, Western blot/Gelatin zymography: 2 | Enzyme linked immunosorbent assay (ELISA), Western blot, Gelatin zymography |
| Lassig et al., 2017 | Patient (surgical wound) | Univeristy medical center | 20 | Radical neck dissection | Closed-suction drainage device | N/A | N/A | No | -80°c | 4 | Once every 8 hours | 7 | Electrochemiluminescent multiarray |
| Lassig et al., 2018 | Patient (surgical wound) | Hospital | 28 | Neck dissection surgical wound | Closed-suction drainage device | N/A | N/A | No | -80°c | 1 | N/A | 14 | Bead based multiplex immunoassay, Enzyme linked immunosorbent assay (ELISA) |
| Lassig et al., 2019 | Patient (surgical wound) | Hospital | 28 | Open head and neck surgical wound | Closed-suction drainage device | N/A | N/A | Yes | -80°c | 1 | N/A | 15 | Bead based multiplex immunoassay, Enzyme linked immunosorbent assay (ELISA) |
| Latijnhouwers et al., 1998 | Patient (open wound) | Hospital | 6 | Venous leg ulcer | Aspiration through occlusive dressing | N/A | N/A | Yes | -20°c | 1 | N/A | 2 | Fluorometric assay, Western blot |
| Lauer et al., 2000 | Patient (open wound), Patient (surgical wound) | Unknown | 25 | Venous leg ulcer, Skin graft donor site, Mastectomy surgical wound | Wound drainage device, Collection from beneath occlusive dressing | N/A | N/A | Yes | -80°c | 1 | N/A | ELISA'Western blot: 1, Spectrophotometry/Colometric assay: 2 | Enzyme linked immunosorbent assay (ELISA), Western blot, Spectrophotometry, Colometric assay |
| Lehnhardt et al., 2005 | Patient (open wound) | Unknown | 11 | Second degree burn | Syringe collection from cutaneous vinyl wound chambers | 3.26 | 6.23 | Yes | -82°c | 6 | Once every 8 hours | Nephelometry: 4, Protein microarray: 1 | Nephelometry, Protein microarray |
| Leiblein et al., 2020 | Patient (surgical wound) | Unknown | 20 | Traumatic bone fracture surgical wound | Closed-suction drainage device | N/A | N/A | Yes | -80°c | 1 | N/A | 2 | Enzyme linked immunosorbent assay (ELISA) |
| Li et al., 2019 | Patient (open wound) | Hospital | 139 | Diabetic foot ulcer, Burn wound | Levine swab technique | N/A | N/A | Yes | -20°c | 1 | N/A | ELISA: 2, Cytokine array: 80 | Enzyme linked immunosorbent assay (ELISA), Cytokine array |
| Ligi et al., 2016 | Patient (open wound) | Hospital | 34 | Venous leg ulcer | Extraction from cotton gauze | N/A | N/A | Yes | -80°c | 1 for each wound stage (inflamed and granulated) where possible | N/A | 13 | Bead based multiplex immunoassay |
| Ligi et al., 2017 | Patient (open wound) | Hospital | 30 | Venous leg ulcer | Extraction from cotton gauze | N/A | N/A | Yes | -80°c | 1 | N/A | 4 | Bead based multiplex immunoassay |
| Lisboa et al., 2013 | Patient (open wound) | Unknown | 75 | Combat-related extremity wound, Bilateral lower-extremity amputation wounds | Negative pressure wound therapy cannister | 30 | N/A | No | -80°c | Varied (until delayed primary closure) | Before each wound debridement | 23 | Bead based multiplex immunoassay |
| Lisowska et al., 2016 | Patient (surgical wound) | Unknown | 23 | Total knee arthroplasty | Wound drainage device | 4.5 | N/A | No | -70°c | 1 | N/A | 1 | Immunoassay |
| Liu et al., 2014 | Patient (open wound) | Hospital | 20 | Pressure ulcer, Surgical incision, Injured soft tissue, Pilonidal abscess, Chronic fistula | Negative pressure wound therapy cannister | N/A | N/A | Unknown | N/A | Unknown | Once a day | 2 | Unknown |
| Lohmann et al., 2017 | Patient (open wound) | Unknown | 6 | Venous leg ulcer | Collection from beneath occlusive dressing | N/A | N/A | Yes | Unknown | 1 | N/A | 6 | Matrix‐assisted laser desorption/ionization–time of flight (MALDI-TOF) |
| Lucas et al., 2018 | Patient (surgical wound) | Univeristy medical center | 20 | Mastectomy surgical wound | Closed-suction drainage device | N/A | N/A | Yes | -70°c | 4 | Collected at 24, 48, 72 and 96 hours post-operatively | 27 | Bead based multiplex immunoassay |
| Lundqvist et al., 2004 | Patient (open wound), Patient (surgical wound) | Hospital | 21 | Venous leg ulcer, Mastectomy surgical wound | Wound drainage device, Aspiration from beneath an occlusive dressing | N/A | N/A | Yes | -20°c | 1 | N/A | 1 | Enzyme linked immunosorbent assay (ELISA), Western blot |
| Mateo et al., 1994 | Patient (surgical wound) | Unknown | 4 | Mastectomy surgical wound | Closed-suction drainage device | N/A | N/A | Yes | -80°c | 7 | Fluids were collected 8, 16, 24, 48, 72, 96 and 120 hours post-operation. | 1 | Bioassay |
| Matsuoka and Grotendorst, 1989 | Patient (surgical wound) | Unknown | 6 | Mastectomy surgical wound | Wound drainage device | N/A | N/A | Yes | -20°c | 7 | Collected daily for 7 days | 1 | Western blot, Bioassay |
| McCarthy et al., 1996 | Patient (open wound) | Hospital | 45 | Partial thickness burn | Aspiration | N/A | N/A | No | -80°c | 1 | N/A | 1 | Enzyme linked immunosorbent assay (ELISA), Western blot, Liquid Chromatography Mass Spectrometry (LCMS), HiTrap Protein A HP column, Heparin affinity chromatography, Radioreceptor assay |
| McDaniel et al., 2011 | Healthy volunteer | Other | 18 | Artificially induced blister | Aspiration | N/A | N/A | No | -80°c | 1 | Fluid was aspirated from four of the blisters at 12 hours postblistering and the other four at 24 hours. | 1 | Protein microarray |
| McDaniel et al., 2017 | Patient (open wound) | Univeristy medical center | 35 | Venous leg ulcer | Aspiration from beneath an occlusive dressing | N/A | N/A | No | -80°c | 3 | Collected on day 0, day 28 and day 56 | 1 | Enzyme linked immunosorbent assay (ELISA), Immunocapture assay |
| McInnes et al., 2014 | Patient (open wound) | Hospital | 33 | Venous leg ulcer, Diabetic foot ulcer | Elution from dressing in buffer | N/A | N/A | Yes | N/A | 1 | N/A | Protein microarray: 18, ELISA: 3, Fluorometric assay: 1, Gelatin zymography: 2 | Protein microarray, Enzyme linked immunosorbent assay (ELISA), Fluorometric assay, Gelatin zymography |
| Meesters et al., 2018 | Healthy volunteer | Univeristy medical center | 49 | Artificially induced blister | Syringe collection from plate attached to wound | N/A | N/A | Yes | -80°c | Varied: 2/3 | Collected at 3,6 and 22 hours | 7 | Multiplex ELISA array |
| Mendez et al., 1999 | Patient (open wound) | Univeristy medical center | 4 | Venous leg ulcer | Withdrawal from dressing using syringe | 16.5 | 6 | Yes | -70°c | Unknown | Unknown | 1 | Enzyme linked immunosorbent assay (ELISA), X-gal–based staining |
| Metcalf et al., 2019 | Patient (open wound) | Hospital | 120 | Venous leg ulcer, Diabetic foot ulcer, Pressure ulcer, Mixed vessel disease ulcer, Arterial ulcer, Traumatic wound, Oncologic ulcer, Amputation wound | Swab technique | 10 | N/A | No | 4°c | 1 | N/A | 3 | Absorbance assay |
| Mikhal'chik et al., 2009 | Patient (open wound) | Hospital | 28 | Full thickness burn | Extraction from filter paper | N/A | N/A | Yes | N/A | 1 | N/A | 4 | Unknown |
| Mikhal'chik et al., 2009 | Patient (open wound) | Unknown | 4 | Full thickness burn | Extraction from filter paper | N/A | N/A | Unknown | N/A | Varied | Collection at 1-3 day intervals | ELISA: 27, Spectrophotometry: 1 | Enzyme linked immunosorbent assay (ELISA), Spectrophotometry |
| Minematsu et al., 2013 | Patient (open wound) | Hospital | 2 | Pressure ulcer | Extraction from nitrocellulose membrane | N/A | N/A | Yes | N/A | 1 | N/A | 1 | Immunostaining |
| Moor et al., 2009 | Patient (open wound) | Wound healing center | 4 | Venous leg ulcer | Extraction from filter paper | 0.125 | 50 | No | -80°c | 3 | Unknown | Fluorometric assay: 3, ELISA/Western blot/Immunocapture assay: 1, Gelatin zymography: 2, Multiplex ELISA array: 21 | Fluorometric assay, Enzyme linked immunosorbent assay (ELISA), Western blot, Gelatin zymography, Multiplex ELISA array, Immunocapture assay |
| Morelli et al., 2011 | Patient (surgical wound) | Dental clinic | 44 | Bilateral mucogingival defect | Extraction from paper strips | N/A | N/A | No | -80°c | 16 | Collected at the 4 corners of the surgical site on weeks 1, 2, 3 and 4 after surgery | 8 | Multiplex ELISA array |
| Moseley et al., 2004 | Patient (open wound) | Hospital | 22 | Venous leg ulcer, Pilonidal abscess, Hidradenitis suppurativa | Extraction from filter paper | 1 | Hidradenitis suppurativa/Pilonidal abscess: 1.476, Venous leg ulcer: 0.644 | No | -20°c | 1 | N/A | N/A | Western blot, Spectrophotometry, SDS-PAGE |
| Moues et al., 2008 | Patient (open wound), Patient (surgical wound) | Unknown | 33 | Pressure ulcer, Surgical wound, Unknown chronic wound, Unknown acute wound | Elution from filter paper in buffer | 1 | N/A | Yes | -20°c | 10 (if possible) | Collected daily for up to 10 days | ELISA: 2, Turbidimeter: 1 | Enzyme linked immunosorbent assay (ELISA), Turbidimeter |
| Muller et al., 2008 | Patient (open wound) | Hospital | 16 | Diabetic foot ulcer | Extraction from paper strips | 1 | N/A | Yes | N/A | 12 | 2 samples collected at weeks 0, 1, 2, 4, 8 and 12. | ELISA: 3, Gelatin zymography: 2 | Enzyme linked immunosorbent assay (ELISA), Gelatin zymography |
| Mwaura et al., 2006 | Patient (open wound) | Wound healing center | 40 | Venous leg ulcer | Withdrawal from dressing using micropipette | N/A | N/A | No | -80°c | 1 | N/A | 3 | Enzyme linked immunosorbent assay (ELISA) |
| Nissen et al., 1998 | Patient (surgical wound) | Unknown | 14 | Mastectomy surgical wound, Neck dissection surgical wound | Closed-suction drainage device | N/A | 17 | Yes | -70°c | Varied | Collected 6 hours after surgery and then daily until patients were discharged or drains were removed | 2 | Enzyme linked immunosorbent assay (ELISA) |
| Nissen et al., 2003 | Patient (open wound), Patient (surgical wound) | Univeristy medical center | 16 | Skin graft donor site, Burn blister, Mastectomy surgical wound, Mammoplasty surgical wound, Cystectomy surgical wound, Heminephrectomy surgical wound | Closed-suction drainage device, Aspiration from beneath an occlusive dressing, Aspiration | N/A | N/A | Yes | -70°c | 1 | N/A | 1 | Enzyme linked immunosorbent assay (ELISA) |
| Nuutila et al., 2013 | Patient (surgical wound) | Hospital | 13 | Skin graft donor site | Negative pressure wound therapy cannister, Aspiration from beneath an occlusive dressing | 2.5 or 13.7 | N/A | Unknown | N/A | 3 | 1st, 2nd and 3rd post-operative days | 1 | Unknown |
| Nwomeh et al., 1998 | Healthy volunteer | Unknown | 20 | Full thickness skin biopsy | Aspiration through occlusive dressing | N/A | N/A | Yes | -20°c | 6 | On days 1,2,3,4,7 and 8 after admission. | 3 | Enzyme linked immunosorbent assay (ELISA) |
| Nwomeh et al., 1999 | Healthy volunteer, Patient (open wound), Patient (surgical wound) | Hospital | 37 | Venous leg ulcer, Pressure ulcer, Mastectomy surgical wound, Full thickness skin biopsy, Flap surgery wound | Closed-suction drainage device, Aspiration through occlusive dressing | N/A | N/A | Yes | -20°c | Open wounds: 6, Surgical wounds: 4 Healthy volunteers: 6 | Open wounds: collected on days 1, 2, 3, 4, 7, and 8. Surgical wounds: collected daily on postoperative days 1–4. healthy volunteers: collected on days 1, 2, 3, 4, 7, and 8. | SDS-PAGE: 2, ELISA: 3 | Enzyme linked immunosorbent assay (ELISA), SDS-PAGE |
| Ono et al., 1995 | Patient (open wound) | Hospital | 12 | Partial thickness burn | Direct syringe collection | 2.5 | N/A | No | -70°c | 1 | N/A | 9 | Enzyme linked immunosorbent assay (ELISA) |
| Ono et al., 1995 | Patient (surgical wound) | Unknown | 24 | Skin graft donor site | Aspiration through occlusive dressing | 2.5 | N/A | No | -70°c | 1 | N/A | 9 | Enzyme linked immunosorbent assay (ELISA) |
| Oono et al., 1997 | Patient (open wound), Patient (other) | Unknown | 13 | Pressure ulcer, Pilonidal abscess, Blister | Direct syringe collection, Extraction from cotton gauze | 3 | N/A | Yes | -20°c | 1 | N/A | 1 | Spectrophotometry, Peptide substrate activity assay |
| Ortega et al., 2000 | Patient (open wound) | Burn center | 5 | Partial thickness burn, Full thickness burn | Aspiration | N/A | N/A | Yes | -70°c | 1 | N/A | 1 | Enzyme linked immunosorbent assay (ELISA) |
| Pajulo et al., 1999 | Patient (surgical wound) | Hospital | 75 | Surgical wound | Cellstick device | 10 | N/A | Yes | N/A | 1 | N/A | 2 | Enzyme linked immunosorbent assay (ELISA) |
| Pallua and Ulrich, 2003 | Patient (surgical wound) | Burn center | 30 | Flap surgery wound | Wound drainage device | N/A | N/A | Yes | -80°c | 3 | At 12, 24 and 36 hours post-operatively | 2 | Enzyme linked immunosorbent assay (ELISA) |
| Palolahti et al., 1993 | Patient (open wound), Patient (surgical wound) | Unknown | 26 | Venous leg ulcer, Skin graft donor site | Aspiration from beneath an occlusive dressing, Direct collection with glass microcapillary | Venous leg ulcer: 0.06 | N/A | Yes | -20°c | Open wound: Up to 3, Surgical wound: Unknown | Open wound: The first specimen was usually taken on the1st or 2nd day of the treatment period and in 5 patients two or three separate samples were collected on different days. Surgical wound: Collected daily until exudation ceased or the dressing detached | Western blot: 1, Casein zymography/Radial immunodiffusion: 2 | Western blot, Casein zymography, Radial immunodiffusion |
| Pan et al., 2012 | Patient (open wound) | Hospital | 87 | Partial thickness burn | Aspiration | N/A | N/A | Unknown | N/A | 1 | N/A | ELISA: 5, Cytokine arrary: 6 | Enzyme linked immunosorbent assay (ELISA), Cytokine array |
| Pellegrini et al., 2017 | Patient (surgical wound) | Unknown | 32 | Intrabony periodontal defect, Horizontal periodontal defect | Extraction from paper strips | N/A | N/A | No | -20°c | 4 | Collected 3–5, 7, 14 and 21 days after surgery. | 9 | Multiplex ELISA array |
| Petrlova et al., 2017 | Patient (surgical wound) | Unknown | 2 | Surgical wound | Closed-suction drainage device | N/A | N/A | Yes | -20°c | 2 | Once 24 hours after surgery and once 24-48 hours after surgery. | 1 | Western blot |
| Piatkowski et al., 2012 | Patient (open wound) | Hospital | 10 | Pressure ulcer | Squeezing dressing applied to wound | N/A | N/A | No | -80°c | 6 | Collected at the start of the study, before treatment (day 0), and on days 3, 7, 14 and 21. | ELISA: 4, Peptide substrate activity assay: 1 | Enzyme linked immunosorbent assay (ELISA), Peptide substrate activity assay |
| Picardo et al., 1992 | Patient (surgical wound) | Hospital | 18 | Laminectomy surgical wound, Brain aneurysm surgical wound, Disk protrusion surgical wound, Submandibular cyst, Hip replacement surgical wound, Facial surgery wound, Molar removal wound, Branchial cyst surgical wound | Closed-suction drainage device | N/A | N/A | Yes | N/A | 1 | N/A | 1 | Cell migration assay |
| Powerski et al., 2011 | Patient (surgical wound) | Unknown | 16 | Musculoskeletal surgical wound | Closed-suction drainage device | N/A | N/A | Yes | -80°c | 3 | Obtained 3, 8, and 24 h postoperatively | ELISA: 1, Cytometric bead array: 2 | Enzyme linked immunosorbent assay (ELISA), Cytometric bead array |
| Prager et al., 1991 | Patient (open wound) | Univeristy medical center | 8 | Burn wound | Collection in sterile plastic sleeve | N/A | N/A | Yes | N/A | 1 | N/A | 1 | Spectrophotometry, Radial immunodiffusion |
| Prager et al., 1994 | Patient (open wound) | Unknown | 19 | Burn wound | Collection in bag | N/A | N/A | Unknown | N/A | 1 | N/A | 2 | Spectrophotometry |
| Prager et al., 1994 | Patient (open wound) | Univeristy medical center | 19 | Burn wound | Direct syringe collection, Collection in bag | N/A | N/A | Yes | N/A | 1 | N/A | Spectrophotometry/Immunocapture assay: 1, Radial immunodiffusion: 3 | Spectrophotometry, Immunocapture assay, Radial immunodiffusion |
| Pukstad et al., 2010 | Patient (open wound) | Hospital | 8 | Venous leg ulcer | Elution from dressing in buffer under constant agitation | N/A | N/A | No | -80°c | 4 if possible | Collected at 14 day intervals for up to 8 weeks or until healing occurred. | ELISA: 1, Cytokine array: 12 | Enzyme linked immunosorbent assay (ELISA), Cytokine array |
| Raffetto et al., 2006 | Patient (open wound) | Unknown | 8 | Venous leg ulcer | Withdrawal from dressing using syringe | N/A | N/A | Yes | -70°c | 1 | N/A | 1 | Western blot |
| Rakmanee et al., 2010 | Patient (surgical wound) | Dental clinic | 15 | Periodontal surgical wound | Extraction from paper strips | 4.2 | N/A | No | -70°c | 4 | All at once | 8 | Bead based multiplex immunoassay |
| Rao et al., 1995 | Patient (open wound), Patient (surgical wound) | Unknown | 13 | Venous stasis ulcer, Surgical wound, Skin graft donor site | Closed-suction drainage device, Aspiration from beneath an occlusive dressing, Expressed mechanically from wound dressing | N/A | 27.5 | If possible | -80°c | 1 | N/A | Western blot: 2, Spectrophotometry/Trypsin Affinity Chromatography: 1 | Western blot, Spectrophotometry, Trypsin Affinity Chromatography |
| Rasmussen et al., 1992 | Patient (open wound) | Unknown | 14 | Venous leg ulcer | Injection and withdrawal of sterile water from wound | N/A | N/A | Unknown | N/A | 15 | On 3 alternate days during treatment period (Weeks 1, 3 and 5) and on 2 days during washout periods (Weeks 2, 4 and 6) | 2 | Radioimmunoassay |
| Rauten et al., 2016 | Patient (surgical wound) | Unknown | 19 | Gingivectomy wound | Extraction from paper strips | 0.1 | N/A | No | -20°c | 6 | Samplings were done 1 hour before gingivectomy and at 24, 72, and 120 hours and 1 and 2 weeks after gingivectomy. | 2 | Enzyme linked immunosorbent assay (ELISA) |
| Rayment et al., 2008 | Patient (open wound) | Nursing facility | 6 | Venous leg ulcer | Collection of wound washout | 1 | N/A | Yes | -80°c | 1 | N/A | 2 | Collagen zymography |
| Rayment et al., 2008 | Patient (open wound) | Nursing facility | 12 | Venous leg ulcer, Blister | Aspiration, Collection of wound washout | Venous leg ulcer: 1 | N/A | Yes | -80°c | 1 | N/A | ELISA: 1, Collagen zymography: 4 | Enzyme linked immunosorbent assay (ELISA), Collagen zymography |
| Reiss et al., 2009 | Patient (open wound) | Unknown | 20 | Burn blister | Aspiration | N/A | N/A | No | -70°c | 1 | N/A | 2 | Western blot, Gelatin zymography |
| Rennekampff et al., 1997 | Patient (open wound), Patient (surgical wound) | Burn center | 28 | Skin graft donor site, Burn blister | Aspiration from beneath an occlusive dressing, Aspiration | N/A | N/A | No | -70°c | Burn wound: 1, Graft wound: Unknown | Burn wound: N/A, Graft wound: Collected on post-operative day 1 and then every 24 hours until no further fluid could be collected | 2 | Enzyme linked immunosorbent assay (ELISA) |
| Rennekampff et al., 2000 | Patient (open wound), Patient (surgical wound) | Univeristy medical center | 43 | Skin graft donor site, Burn blister | Direct syringe collection, Aspiration from beneath an occlusive dressing | Burn blister: 1.5 | N/A | Yes | -70°c | Burn blister: 1 , Skin graft donor sites: Varied (until no more fluid could be obtained) | Burn blister: N/A, Skin graft donor sites: Every day (until no more fluid could be obtained) | 3 | Enzyme linked immunosorbent assay (ELISA) |
| Robson and Heggers, 1981 | Patient (open wound) | Burn center | 10 | Frostbite blister | Aspiration | N/A | N/A | No | Unknown | 1 | N/A | Fluorometric assay: 2, Automated biochemical analyzer: 5, Radial immunodiffusion: 6, Immunoelectrophoresis: 1 | Fluorometric assay, Automated biochemical analyzer, Radial immunodiffusion, Immunoelectrophoresis |
| Rohde et al., 2010 | Patient (surgical wound) | Hospital | 24 | Mammoplasty surgical wound | Closed-suction drainage device | N/A | N/A | No | -80°c | 15 | Wound fluid samples were collected 1 hour postoperatively for the first 6 hours, and on the first postoperative morning before drain removal (at 15 to 24 hours postoperatively). | 4 | Enzyme linked immunosorbent assay (ELISA) |
| Rohde et al., 2015 | Patient (surgical wound) | Hospital | 32 | TRAM Flap Breast Reconstruction | Closed-suction drainage device | 15.25 | N/A | No | -80°c | Unknown | Collection started after 1 hour post-operation and continued hourly for the first 6 hours and every 6-12 hour intervals thereafter. | 4 | Enzyme linked immunosorbent assay (ELISA) |
| Ruf et al., 2017 | Patient (open wound) | Hospital | 10 | Venous leg ulcer | Squeezing sponges applied to wound | 0.06 | N/A | No | -20°c | Varied (1-3) | Wound fluid was collected at day 0 of recruitment and, if possible, 6 weeks later. | ELISA: 1, Gelatin zymography: 2 | Enzyme linked immunosorbent assay (ELISA), Gelatin zymography |
| Sabino et al., 2018 | Patient (open wound) | Univeristy medical center | 10 | Unknown chronic wound | Extraction from dressing | N/A | N/A | No | -80°c | 4 | Every 2 days | 2667 | iTRAQ Mass Spectrometry |
| Sadler et al., 2012 | Patient (open wound) | Hospital | 20 | Venous leg ulcer | Aspiration from beneath an occlusive dressing | N/A | N/A | No | -80°c | 1 | N/A | Fluorometric assay: 3, ELISA: 1 | Fluorometric assay, Enzyme linked immunosorbent assay (ELISA) |
| Saglam et al., 2014 | Patient (open wound) | Dental clinic | 30 | Periodontal pocket | Extraction from paper strips | 0.65 | N/A | Yes | -80°c | 4 | At baseline and at 1, 3 and 6 months after treatment | 6 | Enzyme linked immunosorbent assay (ELISA) |
| Saravanan et al., 2017 | Patient (open wound), Patient (surgical wound) | Unknown | 4 | Venous leg ulcer, Mastectomy surgical wound | Collection from beneath occlusive dressing, Wound drainage device | N/A | N/A | Yes | -20°c | 1 | N/A | 1 | Liquid Chromatography Mass Spectrometry (LCMS) |
| Sarment et al., 2006 | Patient (surgical wound) | Dental clinic | 47 | Periodontal pocket | Extraction from paper strips | N/A | N/A | No | -20°c | 6 | Wound fluid was collected at baseline, weeks 3, 6, 12, 18 and 24 after re-constructive surgery. | 1 | Radioimmunoassay |
| Scherer et al., 2016 | Patient (surgical wound) | Unknown | 23 | Lumpectomy surgical wound | Wound drainage device | 37.35 | N/A | Yes | -80°c | 1 | N/A | 1 | Enzyme linked immunosorbent assay (ELISA) |
| Scherzad et al., 2019 | Patient (surgical wound) | Univeristy medical center | 7 | Neck dissection surgical wound | Vacuum drainage device | N/A | N/A | Yes | N/A | 1 | N/A | Western blot: 1, Dot blot: 22 | Western blot, Dot blot |
| Scherzed et al., 2011 | Patient (surgical wound) | Univeristy medical center | 0 | Neck dissection surgical wound | Vacuum drainage device | N/A | N/A | Yes | -80°c | Unknown | Unknown | 14 | Dot blot, Flow cytometry |
| Schiffer et al., 2015 | Patient (open wound), Patient (surgical wound) | Hospital | 14 | Venous leg ulcer, Diabetic foot ulcer, Pressure ulcer, Arterial ulcer, Amputation wound, Traumatic ulcer | Swab technique | N/A | N/A | Unknown | N/A | 1 | N/A | 1 | Spectrophotometry, Bioassay, Enzyme‐dependent dye trapping system |
| Schmidtchen et al., 2002 | Patient (open wound), Patient (surgical wound) | Unknown | 5 | Venous leg ulcer, Mastectomy surgical wound | Wound drainage device, Collection from beneath occlusive dressing | Venous leg ulcer: 0.35 | N/A | Yes | -20°c | 1 | N/A | 1 | SDS-PAGE, Zymography |
| Schmidtchen, 1999 | Patient (open wound) | Unknown | 5 | Venous leg ulcer | Extraction from filter paper | 10 | 23 | Yes | -20°c | 1 | N/A | 1 | Western blot |
| Schmidtchen, 2000 | Patient (open wound) | Hospital | 12 | Venous leg ulcer | Extraction from filter paper, Collection from beneath occlusive dressing | 0.6 | N/A | Yes | -20°c | 1 | N/A | 15 | Western blot |
| Schmohl et al., 2012 | Patient (open wound), Patient (surgical wound) | Hospital | 95 | Diabetic foot ulcer, Hernia wound | Wound drainage device, Levine swab technique, Aspiration through occlusive dressing | Foot ulcer: 0.04 | N/A | Yes | -80°c | 1 | N/A | 20 | Multiplex ELISA array, LDS-PAGE |
| Schonfelder et al., 2005 | Patient (open wound) | Unknown | 1 | Venous leg ulcer | Negative pressure wound therapy cannister | N/A | 6.4 | No | -80°c | 1 | N/A | 1 | Fluorometric assay, Enzyme linked immunosorbent assay (ELISA) |
| Segatto et al., 2014 | Patient (surgical wound) | Specialised medical center | 0 | Lumpectomy surgical wound | Wound drainage device | N/A | N/A | Yes | -80°c | 1 | N/A | 11 | Western blot, Immunoprecipitation (IP) |
| Segatto et al., 2014 | Patient (surgical wound) | Specialised medical center | 0 | Lumpectomy surgical wound | Wound drainage device | N/A | N/A | Yes | -80°c | 1 | N/A | 10 | Western blot |
| Senet et al., 2003 | Patient (open wound) | Hospital | 13 | Venous leg ulcer | Aspiration from beneath an occlusive dressing | N/A | N/A | Yes | -80°c | 4 | Collected on entry to the study and then every 4 weeks for 12 weeks. | 4 | Enzyme linked immunosorbent assay (ELISA) |
| Serena et al., 2016 | Patient (open wound) | Wound healing center | 290 | Venous leg ulcer, Diabetic foot ulcer, Pressure ulcer, Surgical wound, Arterial ulcer, Traumatic wound | Serena swab technique | 0.11 | N/A | No | -70°c | 1 | N/A | 1 | Fluorometric assay, Spectrophotometry |
| Serra et al., 2013 | Patient (open wound), Patient (surgical wound) | Unknown | 51 | Venous leg ulcer, Orthopedic surgical wound | Wound drainage device, Aspiration from beneath an occlusive dressing | N/A | N/A | Yes | -80°c | Open wounds: 3, Surgical wounds: 2 | Open wounds: Once at admission and then once at weeks 4 and 8 after admission. Surgical wounds: Once at the time of surgery and once 24 hours after surgery | 2 | Enzyme linked immunosorbent assay (ELISA) |
| Sexton et al., 2014 | Patient (surgical wound) | Plastic surgery clinic | 33 | Abdominoplasty surgical wound | Wound drainage device | N/A | N/A | No | -80°c | 3 | Collected at days 2, 3 and 4 after surgery. | 5 | Enzyme linked immunosorbent assay (ELISA) |
| Shakespeare et al., 1978 | Patient (open wound) | Burn center | 4 | Burn blister | Aspiration | N/A | 62.5 | No | -20°c | 1 | N/A | SDS-PAGE: 3, Peptide substrate activity assay: 1, Rocket immunoelectrophoresis: 9 | SDS-PAGE, Peptide substrate activity assay, Rocket immunoelectrophoresis |
| Smeets et al., 2008 | Patient (open wound) | Hospital | 27 | Venous leg ulcer | Extraction from dressing | N/A | N/A | No | -80°c | 1 | N/A | 1 | Fluorometric assay, Enzyme linked immunosorbent assay (ELISA), Spectrophotometry, Peptide substrate activity assay |
| Smith and Hoffman, 2005 | Patient (open wound) | Wound healing center | 6 | Venous leg ulcer | Elution from dressing in buffer | N/A | N/A | Yes | -70°c | 1 | N/A | 4 | Western blot, Immunoprecipitation (IP) |
| Smith et al., 2015 | Healthy volunteer | Other | 16 | Artificially induced blister | Direct syringe collection | 1 | N/A | Unknown | N/A | 1 | N/A | 5 | Bead based multiplex immunoassay |
| Stacey and Farrokhyar, 2020 | Patient (open wound) | Hospital | 24 | Venous leg ulcer | Aspiration from beneath an occlusive dressing | N/A | N/A | No | -80°c | 10 (if possible)- total of 121 samples | Once a week (if possible) | 52 | Multiplex ELISA array |
| Stanley et al., 2008 | Patient (open wound) | Hospital, Dental clinic | 43 | Diabetic foot ulcer, Periodontal pocket | Extraction from paper strips | 0.05 | N/A | No | -80°c | Foot ulcer: 3, periodontal pocket: 2 | All at once | 1 | Western blot |
| Starlinger et al., 2012 | Patient (surgical wound) | Hospital | 50 | Colorectal cancer surgical wound | Wound drainage device | N/A | N/A | Yes | -70°c | 3 | Collected on post-operative days 1-3 | 1 | Enzyme linked immunosorbent assay (ELISA), Immunoprecipitation (IP) |
| Steinsträßer et al., 2010 | Patient (open wound) | Unknown | 6 | Unknown chronic wound, Unknown acute wound | Centrifugation of PVA sponges applied to wound | 2 | 35 | Yes | -80°c | 1 | N/A | ELISA: 1, SDS-PAGE: 20, LCMS: 355 | Enzyme linked immunosorbent assay (ELISA), Liquid Chromatography Mass Spectrometry (LCMS), SDS-PAGE |
| Subramaniam et al., 2008 | Patient (open wound), Patient (surgical wound) | Unknown | 20 | Venous leg ulcer, Skin graft donor site, Mastectomy surgical wound | Aspiration from beneath an occlusive dressing, Vacuum drainage device | N/A | Mastectomy surgical wound: 41.7, Skin graft donor site: 65, Venous leg ulcer: 40.9 | Yes | -80°c | 1 | N/A | 3 | Enzyme linked immunosorbent assay (ELISA) |
| Subramanian et al., 1997 | Patient (surgical wound) | Hospital | 0 | Mammoplasty surgical wound | Closed-suction drainage device | N/A | N/A | Yes | -70°c | 3 | Collected at 1 day intervals for 3 consecutive days | 2 | Western blot, Dot blot |
| Tamai et al., 2016 | Patient (open wound) | Hospital | 20 | Malignant fungating wound | Centrifugation of wound dressing | 10 | N/A |  |  |  |  | 2 | Gelatin zymography |
| Tan et al., 2016 | Patient (open wound) | Hospital | 124 | Diabetic foot ulcer, Foot ulcer | Extraction from filter paper | 1 | N/A | Yes | -80°c | 1 | N/A | 1 | Enzyme linked immunosorbent assay (ELISA) |
| Tanzer et al., 2015 | Patient (open wound) | Hospital, Burn center | 66 | Burn blister | Aspiration, Direct collection using capillary pipette | N/A | N/A | Yes | -80°c | 1 | N/A | 1 | Enzyme linked immunosorbent assay (ELISA), Western blot |
| Tao et al., 2015 | Patient (open wound) | Hospital | 32 | Unknown chronic wound | Extraction from filter paper | 0.1 | N/A | No | -80°c | 3 | At admission and then weekly | 4 | Bead based multiplex immunoassay |
| Tarlton et al., 1997 | Patient (open wound), Patient (surgical wound) | Unknown | 24 | Venous leg ulcer, Mastectomy surgical wound, Colectomy surgical wound | Wound drainage device, Collection from beneath occlusive dressing | N/A | N/A | Yes | -80°c | Mastectomy: 6, Colectomy: 5, VLU: Unknown | Mastectomy: 3, 6, 24, 48, 72 and 96 hours, Colectomy: 24, 48, 72, 96 and 120, VLU: Greater than 24 hours between each collection | 2 | Gelatin zymography |
| Tarlton et al., 1999 | Patient (open wound) | Unknown | 26 | Venous leg ulcer | Extraction from filter paper | N/A | N/A | No | -20°c | At least 5 | Collections were taken on at least five occasions over a one year period, with the collection intervals, and the wound treatment, being determined by clinical need. | ELISA/Casein zymography: 1, Gelatin zymography: 2 | Enzyme linked immunosorbent assay (ELISA), Gelatin zymography, Casein zymography |
| Tarnuzzer and Schultz, 1996 | Patient (open wound), Patient (surgical wound) | Unknown | 19 | Mastectomy surgical wound, Unknown chronic wound | Wound drainage device, Aspiration from beneath an occlusive dressing | N/A | N/A | Yes | N/A | Chronic wound: 1, Surgical wound: 7 | Chronic wound: N/A, Surgical wound: Every post-operative day for 7 days | Radioimmunoassay: 4, Azocoll assay: 1, ELISA: 2 | Radioimmunoassay, Azocoll assay, Enzyme linked immunosorbent assay (ELISA) |
| Tokunaga et al., 1993 | Patient (surgical wound) | Hospital | 28 | cholecystectomy surgical wound, Mastectomy surgical wound, Gastrectomy surgery wound | Wound drainage device | N/A | N/A | Unknown | N/A | 3 | Collected on days 1, 3 and 5 after surgery. | ELISA: 1, Radioimmunoassay: 2 | Enzyme linked immunosorbent assay (ELISA), Radioimmunoassay |
| Trengove et al., 1996 | Patient (open wound) | Hospital | 8 | Venous leg ulcer, Mixed vessel disease ulcer | Aspiration from beneath an occlusive dressing | N/A | 39 | Yes | N/A | 2 | 24 hours after admission and after 2 weeks of treatment | Automated biochemical analyzer: 7, Turbidimeter: 3 | Automated biochemical analyzer, Turbidimeter |
| Trengove et al., 2000 | Patient (open wound) | Hospital | 26 | Venous leg ulcer, Mixed vessel disease ulcer | Aspiration from beneath an occlusive dressing | N/A | N/A | Yes | -80°c | 2 | Collected on admission and after 2 weeks. | ELISA: 8, Bioassay: 2 | Enzyme linked immunosorbent assay (ELISA), Bioassay |
| Trengove., 1999 | Patient (open wound), Patient (surgical wound) | Hospital | 33 | Diabetic foot ulcer, Decubitus ulcer, Mixed vessel disease ulcer, Mastectomy surgical wound | Vacuum drainage device, Aspiration from beneath an occlusive dressing | N/A | N/A | No | -80°c | Surgical wounds: Different for each patient. Open wounds: 2 | Surgical wounds: Once a day for up to 7 days. Open wounds: Once within 24 hours of admission and once after 2 weeks during healing phase | ELISA/Gamma scintillation counter: 1, Spectrophotometry/Azocoll assay: 2 | Enzyme linked immunosorbent assay (ELISA), Spectrophotometry, Azocoll assay, Gamma scintillation counter |
| Trostrup et al., 2011 | Patient (open wound) | Wound healing center | 33 | Venous leg ulcer, Pilonidal abscess | Collection from beneath occlusive dressing | N/A | N/A | Yes | -80°c | Chronic wounds: 4, acute wounds: Unknown | Chronic wounds: Collected weekly over a 4 week period, Acute wounds: Unknown | ELISA/Western blot/Gelatin zymography: 1, Protein microarray: 40 | Enzyme linked immunosorbent assay (ELISA), Protein microarray, Western blot, Gelatin zymography |
| Trostrup et al., 2016 | Patient (open wound), Patient (surgical wound) | Wound healing center | 24 | Venous leg ulcer, Diabetic foot ulcer, Skin graft donor site | Fluid squeezed out of dressing using syringe | N/A | N/A | Yes | -80°c | 1 | N/A | Bead based multiplex immunoassay: 3, ELISA: 1 | Bead based multiplex immunoassay, Enzyme linked immunosorbent assay (ELISA) |
| Trostrup et al., 2018 | Patient (open wound), Patient (surgical wound) | Wound healing center | 24 | Venous leg ulcer, Diabetic foot ulcer, Skin graft donor site | Fluid squeezed out of dressing using syringe | N/A | N/A | Yes | -80°c | 1 | N/A | ELISA: 2, Gelatin zymography/Immunocapture assay: 1 | Enzyme linked immunosorbent assay (ELISA), Gelatin zymography, Immunocapture assay |
| Twetman et al., 2018 | Healthy volunteer | Hospital | 10 | Punch biopsy | Extraction from filter paper | 0.15 | 0.82 | Yes | -80°c | 3 | Collected after 2, 5 and 8 days post-biopsy | 6 | Multiplex ELISA array |
| Ulrich et al., 2011 | Patient (open wound) | Hospital | 32 | Diabetic foot ulcer | Extraction from dressing | N/A | N/A | No | -80°c | 9 | Wound exudate was collected from the patients on admission, at days 5 and 14, and at 14-day periods thereafter for 12 weeks. | 1 | Fluorometric assay, Enzyme linked immunosorbent assay (ELISA), Spectrophotometry, Peptide substrate activity assay |
| Ure et al., 1998 | Healthy volunteer | Unknown | 10 | Artificial superficial skin wound | Collection from sterile skin chambers | N/A | N/A | Yes | -20°c | Varied: Up to 9 | Collected on each day for up to 9 days | 7 | Enzyme linked immunosorbent assay (ELISA) |
| Utz et al., 2010 | Patient (open wound) | Specialised medical center | 25 | Combat-related extremity wound | Negative pressure wound therapy cannister | 30 | N/A | No | -80°c | Varied | Collected 2 h following the first surgical debridement and over a 12-h period prior to each subsequent wound debridement (every 48-72 hours) | 5 | Bead based multiplex immunoassay |
| van den Broek et al., 2014 | Patient (open wound), Patient (surgical wound) | Hospital | 28 | Venous leg ulcer, Full thickness burn, Tumor excision surgical wound | Extraction from dressing, Collection from debridement material | N/A | 4.18 | Yes | -80°c | 1 | N/A | 17 | Enzyme linked immunosorbent assay (ELISA) |
| Varelias et al., 2006 | Patient (open wound) | Hospital | 20 | Venous leg ulcer, Diabetic foot ulcer, Arterial ulcer, Vasculitic ulcer | Aspiration | N/A | N/A | Yes | -70°c | 4 | Collected 1–2 days prior to treatment and at day 15, day 29 and day 36. | 2 | Western blot, Gelatin zymography |
| Vogt et al., 1998 | Patient (surgical wound) | Unknown | 16 | Skin graft donor site | Syringe collection from cutaneous vinyl wound chambers | 3 | N/A | Yes | -70°c | Varied (Until wound healed) | Every 24 hours | ELISA: 6, Radioimmunoassay: 3 | Enzyme linked immunosorbent assay (ELISA), Radioimmunoassay |
| Wall et al., 2002 | Patient (open wound), Patient (surgical wound) | Hospital | 22 | Surgical incision, Unknown chronic wound | Extraction from filter paper | N/A | N/A | Yes | N/A | 1 | N/A | Western blot: 1, Gelatin zymography: 2 | Western blot, Gelatin zymography |
| Wallace and Stacey, 1998 | Patient (open wound) | Hospital | 21 | Venous leg ulcer | Aspiration | N/A | N/A | Yes | -80°c | 2 | Collected from the patients ulcers within 24 h of admission to hospital and after 2 wk of regular saline dressings and bed rest. | ELISA: 3, Bioassay: 1 | Enzyme linked immunosorbent assay (ELISA), Bioassay |
| Wang et al., 2015 | Patient (surgical wound) | Hospital | 45 | Mastectomy surgical wound, Mammary benign disease surgical wound | Wound drainage device | 50 | N/A | Yes | -80°c | Varied | Collected from post-operative days 1-4. | 15 | Bead based multiplex immunoassay |
| Wang et al., 2019 | Patient (open wound) | Hospital | 100 | Diabetic foot ulcer | Swab technique | N/A | N/A | No | -20°c | 1 | N/A | 1 | Enzyme linked immunosorbent assay (ELISA) |
| Weckroth et al., 1996 | Patient (open wound), Patient (surgical wound) | Hospital | 16 | Venous leg ulcer, Skin graft donor site | Collection from beneath occlusive dressing, Direct collection with glass microcapillary | N/A | N/A | Yes | -20°c | Chronic wounds: Unknown, Acute wounds: Maximum of 5/6 | Chronic wounds: Before daily treatment, Acute wounds: Collected on several days following operation until the exudation ceased (usually 5-6 days). | Western blot: 4, Spectrophotometry/Gelatin zymography: 2, SDS-PAGE/Peptide substrate activity assay: 1 | Western blot, Spectrophotometry, Gelatin zymography, SDS-PAGE, Peptide substrate activity assay |
| Weckroth et al., 2001 | Patient (open wound), Patient (surgical wound) | Hospital | 15 | Venous leg ulcer, Skin graft donor site | Aspiration from beneath an occlusive dressing | N/A | N/A | Yes | -70°c | Skin graft wound fluid: 3. Chronic wound fluid: 1 | Skin graft wound fluid: On days 1,3 and 5 after surgery. Chronic wound fluid: N/A | 2 | Enzyme linked immunosorbent assay (ELISA), Immunocapture assay |
| Werthen et al., 2004 | Patient (open wound), Patient (surgical wound) | Unknown | 0 | Venous leg ulcer, Mastectomy surgical wound | Wound drainage device, Aspiration from beneath an occlusive dressing | N/A | N/A | Yes | -20°c | 1 | N/A | 2 | Gelatin zymography, SDS-PAGE |
| Wicke et al., 2002 | Patient (surgical wound) | Hospital | 30 | Orthopedic surgical wound | Closed-suction drainage device | N/A | 64 | Yes | -70°c | 4 | Once a day | 4 | Radioimmunoassay |
| Wiegand et al., 2010 | Patient (open wound), Patient (surgical wound) | Hospital | 38 | Venous leg ulcer, Diabetic foot ulcer, Mixed vessel disease ulcer, Ablation wound | Collection of wound washout | 2 | N/A | No | -80°c | 3 | Collected on arrival as well as 7 and 14 days following treatment. | 6 | Enzyme linked immunosorbent assay (ELISA) |
| Wiegand et al., 2017 | Patient (open wound) | Unknown | 36 | Venous leg ulcer | Extraction from paper strips | 0.1 | N/A | No | -80°c | 3 | Collected on arrival as well as 7 and 14 days following treatment. | 5 | Bead based multiplex immunoassay |
| Wiik et al., 2004 | Patient (surgical wound) | Hospital | 24 | Gastrointestinal surgery wound | Syringe collection from a surgical drainage tube | N/A | N/A | No | -20°c | 7 | Collected daily for 7 days | ELISA: 1, Radioimmunoassay: 2 | Enzyme linked immunosorbent assay (ELISA), Radioimmunoassay |
| Wilson et al., 1994 | Patient (open wound) | Hospital | 25 | Partial thickness burn | Aspiration | N/A | 44.7 | No | -20°c | 1 | N/A | Western blot/Dot blot/Peptide substrate activity assay: 1, Radioimmunoassay: 2 | Western blot, Spectrophotometry, Radioimmunoassay, Dot blot, Peptide substrate activity assay |
| Wu et al., 2003 | Patient (surgical wound) | Unknown | 16 | Mastectomy surgical wound | Wound drainage device | 65.65 | N/A | Yes | -80°c | 2 | Collected on day 1 and 4 post-operatively | 2 | Enzyme linked immunosorbent assay (ELISA) |
| Wu et al., 2004 | Patient (surgical wound) | Unknown | 26 | Colorectal cancer surgical wound | Vacuum drainage device | 85.25 | N/A | Yes | -80°c | 2 | Collected on days 1 and 4 | 2 | Enzyme linked immunosorbent assay (ELISA) |
| Wyffels et al., 2010 | Patient (open wound) | Unknown | 17 | Pressure ulcer | Extraction from polyester tipped applicators | 0.5 | N/A | Yes | -80°c | 15 (if possible) | On days 0, 1, 2, 3, 4, 7, 8, 9, 10, 11, 14, 21, 28, 35 and 42 (If possible). | 11 | Liquid Chromatography Mass Spectrometry (LCMS) |
| Wysocki et al., 1993 | Healthy volunteer, Patient (open wound), Patient (surgical wound) | Hospital | 20 | Venous leg ulcer, Artificially induced blister, Mastectomy surgical wound | Wound drainage device, Aspiration from beneath an occlusive dressing | N/A | N/A | Yes | -70°c | 1 | N/A | 2 | Gelatin zymography |
| Wysocki et al., 1999 | Patient (open wound), Patient (surgical wound) | Hospital | 17 | Venous stasis ulcer, Mastectomy surgical wound | Closed-suction drainage device, Withdrawal from dressing using syringe | Mastectomy surgical wound: 17.5 | N/A | Unknown | N/A | Acute wounds: Up to 4, Chronic wounds: Unknown | Acute wounds: Fluid was collected within the first 24 hours after surgery and then every day until either the drains were removed or the patient was discharged, typically 2–4 days after surgery. Chronic wounds: Unknown | Western blot/Zymography: 2, Spectrophotometry/Gelatin zymography/Peptide substrate activity assay: 1 | Western blot, Spectrophotometry, Gelatin zymography, Peptide substrate activity assay, Zymography |
| Wysocki, 1996 | Healthy volunteer, Patient (open wound), Patient (surgical wound) | Hospital | 31 | Venous leg ulcer, Artificially induced blister, Mastectomy surgical wound | Closed-suction drainage device, Aspiration through occlusive dressing, Suction collection | N/A | N/A | Unknown | N/A | Acute wounds: Up to 4, Chronic wounds: Unknown | Acute wounds: Fluid was collected within the first 24 hours after surgery and then every day until either the drains were removed or the patient was discharged, typically 2–4 days after surgery. Chronic wounds: Unknown | Western blot: 1, Gelatin zymography: 2 | Western blot, Gelatin zymography |
| Yager et al., 1996 | Patient (open wound), Patient (surgical wound) | Hospital | 10 | Pressure ulcer, Abdominoplasty surgical wound, Mastectomy surgical wound, Mammoplasty surgical wound | Closed-suction drainage device, Aspiration from beneath an occlusive dressing | N/A | Pressure ulcer: 28.1, Abdominoplasty surgical wound/Mammoplasty surgical wound/Mastectomy surgical wound: 28.7 | Yes | -20°c | Open wounds: 1, Surgical wounds: 3 | Open wounds: N/A, Surgical wounds: Collected daily on days 1-3 | Fluorometric assay/ELISA: 1, Gelatin zymography: 2 | Fluorometric assay, Enzyme linked immunosorbent assay (ELISA), Gelatin zymography, SDS-PAGE |
| Yager et al., 1997 | Patient (open wound), Patient (surgical wound) | Hospital | 15 | Pressure ulcer, Abdominoplasty surgical wound, Venous stasis ulcer | Closed-suction drainage device, Aspiration from beneath an occlusive dressing | N/A | N/A | Yes | -20°c | 1 for all but one surgical patient from which 6 samples were collected | On postoperative days 1, 2, 3, 4, 6, and 9 for one surgical patient | ELISA/Western blot: 2, Spectrophotometry: 1 | Enzyme linked immunosorbent assay (ELISA), Western blot, Spectrophotometry |
| Yao et al., 2014 | Patient (open wound) | Univeristy medical center | 12 | Diabetic foot ulcer | Extraction from filter paper | 0.1 | N/A | No | -80°c | 4 | Once per week | 7 | Bead based multiplex immunoassay |
| Yeoh-Ellerton and Stacey, 2003 | Patient (open wound), Patient (surgical wound) | Hospital | 33 | Venous leg ulcer, Mastectomy surgical wound | Aspiration from beneath an occlusive dressing, Vacuum drainage device | N/A | N/A | Yes | -80°c | Open wound: 2, Surgical wound: 1 | Open wound: Collected on admission to hospital, and after 2 wk of hospitalization. Surgical wound: N/A | 2 | Automated biochemical analyzer |
| Young and Grinnell, 1994 | Patient (open wound) | Unknown | 3 | Partial thickness burn, Full thickness burn | Collection in sterile glove | N/A | Full thickness burn: 17.5, Partial thickness burn: 40 | Yes | -70°c | Varied | Sampling of burn fluid was initiated 4-8 h after injury (day 0) and continued at 48- to 72-h intervals until day 8 for patient 5-1, until day 13 for patient 5-2, and until day 6 for patient 5-4. | Western blot: 1, Gelatin zymography: 2 | Western blot, Gelatin zymography, Azocoll assay |
| Zaleska et al., 2016 | Patient (surgical wound) | Specialised medical center | 44 | Quadrantectomy surgical wound | Percutaneous aspiration | N/A | N/A | Yes | -80°c | 1 | N/A | 1 | Fluorescence-activated cell sorting (FACS) |
| Zang et al., 2016 | Patient (open wound) | Hospital | 39 | Burn blister | Aspiration, Direct collection using capillary pipette | N/A | 51.6 | Yes | -80°c | 1 | N/A | 401 | Liquid Chromatography Mass Spectrometry (LCMS), LDS-PAGE, Filter aided sample preparation (FASP), Isoelectric focusing, Immunodepletion |
| Zhang et al., 2019 | Patient (surgical wound) | Hospital | 199 | Thyroid surgical wound | Closed-suction drainage device | 55.5 | N/A | Unknown | N/A | 2 | Once a day | 1 | Western blot |
| Zhu et al., 2018 | Patient (surgical wound) | Hospital | 30 | Mastectomy surgical wound | Wound drainage device | 5 | N/A | Yes | -80°c | 4 | Collected at 2 hours and on days 1, 3, and 7 postoperatively. | 5 | Enzyme linked immunosorbent assay (ELISA) |
| Zillmer et al., 2011 | Patient (open wound) | Unknown | 23 | Venous leg ulcer | Aspiration from beneath an occlusive dressing, Fluid squeezed out of dressing using syringe | N/A | 55.15 | Yes | -80°c | 1st cohort: 5, 2nd cohort: 2 | 1st cohort: Every 8 hours (3x) then every 24 hours (2x). 2nd cohort: After 1 hour and then after 24 hours. | 4 | Enzyme linked immunosorbent assay (ELISA) |


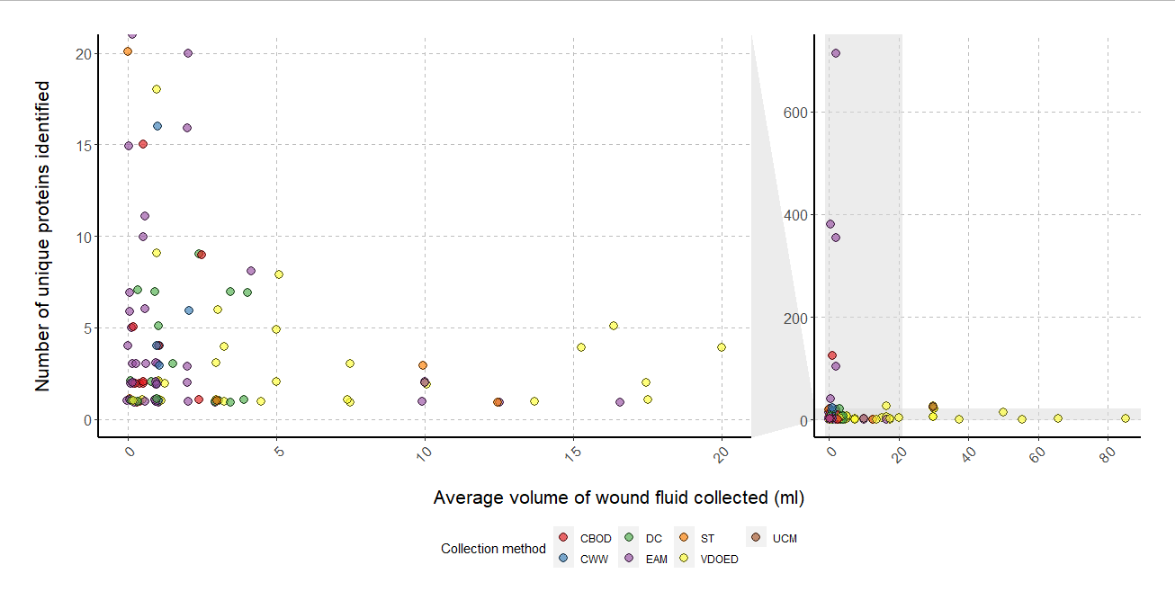


***Figure S1.*** The average volume of wound fluid collected (ml) and the number of unique proteins identified for all included studies.

Each point represents an included study. Some studies recorded separate average wound fluid volumes for each investigated wound type and so were included multiple times. Wound fluid volumes were recorded for 100 of the 280 included studies. *CBOD, Collection beneath an occlusive dressing; CWW, Collection of wound washout; DC, Direct collection; EAM, Extraction from absorbent materials; ST, Swab technique; VDOED, Vacuum, drainage or other external device; UCM, Unknown collection method.*


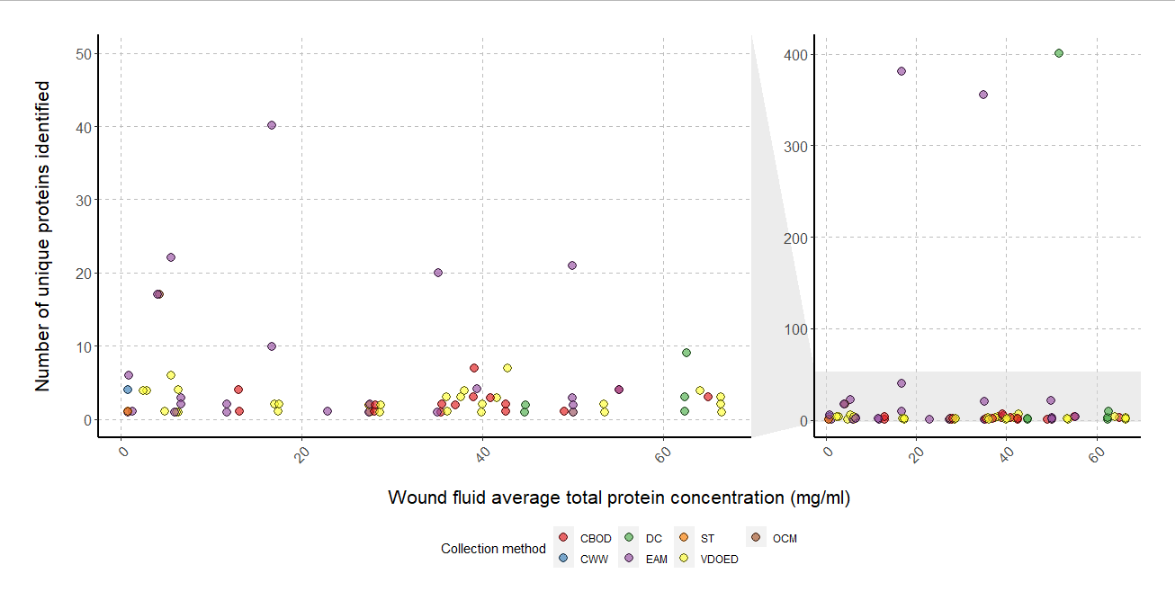


***Figure S2.*** The average total protein concentration of collected wound fluid (mg/ml) and the number of unique proteins identified for all included studies.

Each point represents an included study. Some studies recorded separate average total protein concentrations for each investigated wound type and so were included multiple times. Wound fluid total protein concentrations were recorded for 41 of the 280 included studies. *CBOD, Collection beneath an occlusive dressing; CWW, Collection of wound washout; DC, Direct collection; EAM, Extraction from absorbent materials; ST, Swab technique; VDOED, Vacuum, drainage or other external device; OCM, Other collection methods.*
